# Supplementary material for: Biosynthesis of iridoid sex pheromones in aphids
Source: Proc Natl Acad Sci U S A. 2022 Oct 13;119(42):e2211254119. doi: 10.1073/pnas.2211254119 (PMC9586269; doi:10.1073/pnas.2211254119)
Supplement: Supplementary File [file pnas.2211254119.sapp.pdf]

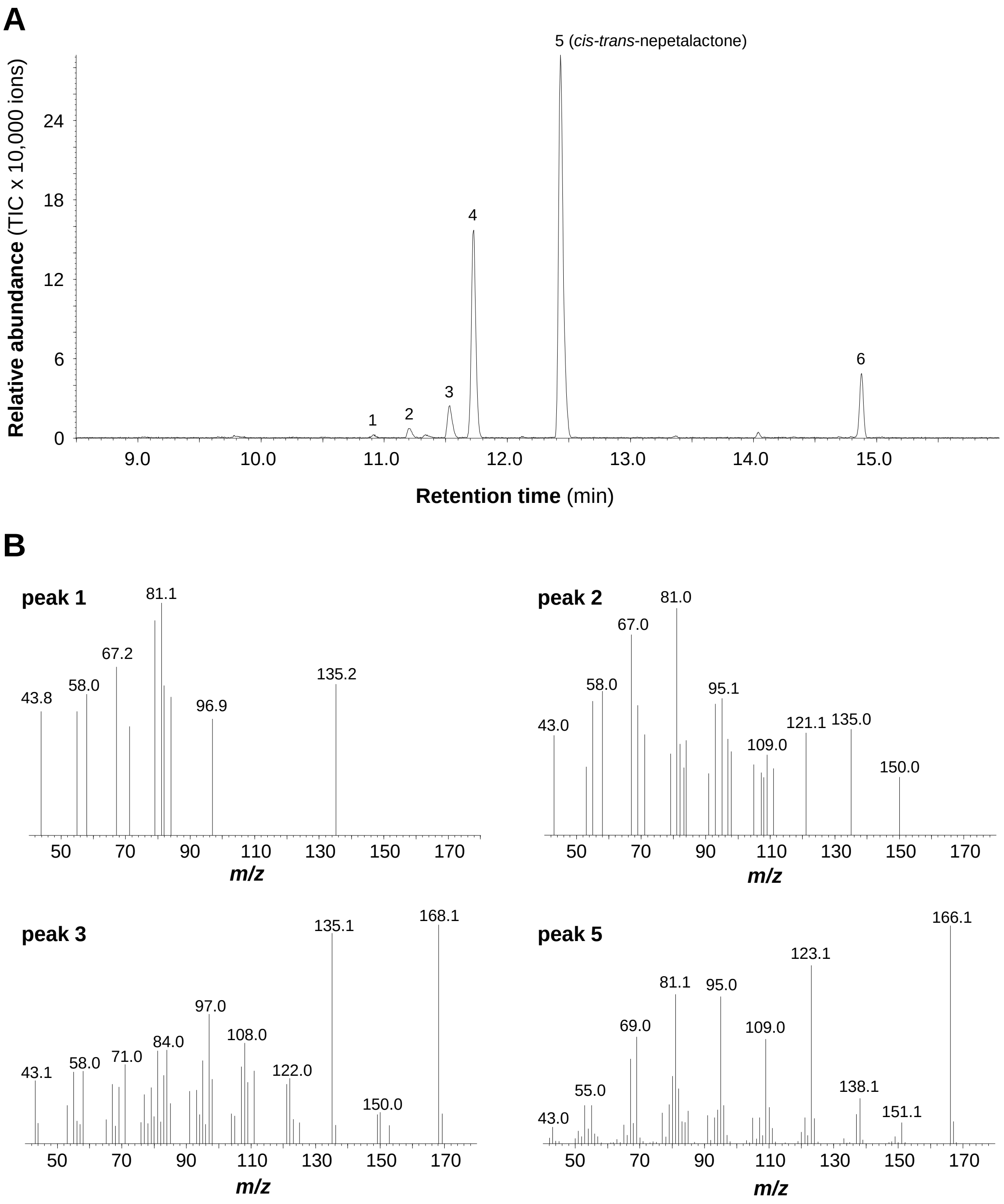

Supplemental Figure S1: Sexual females of the pea aphid *Acyrthosiphon pisum* release volatile iridoids. (A) Asexual *A. pisum* aphids were reared on *Vicia faba* plants in closed cellophane bags under short-day conditions and reduced temperature to stimulate the development of sexual females. Volatiles released from sexual female aphids were collected from the headspace of the plant/aphids using solid-phase microextraction (SPME) and analyzed with gas chromatography-mass spectrometry (GC-MS). 1, unidentified; 2, unidentified; 3, *cis-trans*-nepetalactol; 4, dodecamethylcyclohexasiloxane (contamination); 5, *cis-trans*-nepetalactone; 6, tetradecamethylcycloheptasiloxane (contamination). **(B)** Mass spectra of peaks 1,2,3, and 5 of the chromatogram shown in panel A.

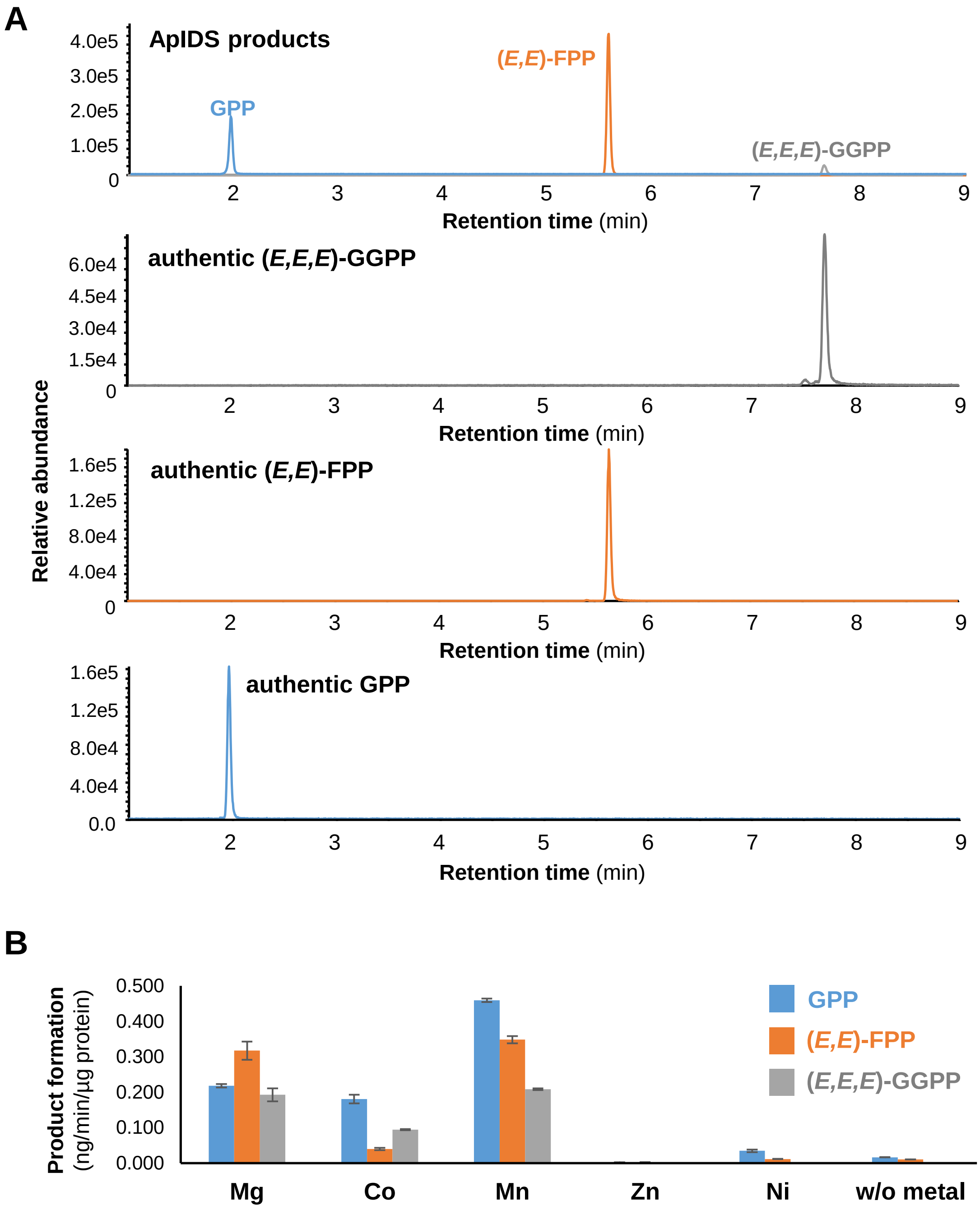

Supplemental Figure S2: The metal ion cofactor influences product specificity of ApIDS. (A) Identification of ApIDS products using authentic standard compounds. N-terminal truncated ApIDS was expressed as N-terminal His-tag-fusion protein in *Escherichia coli*, purified, and incubated with the substrates IPP and DMAPP in the presence of 1 mM $MgCl_2$. Products were analyzed using liquid chromatography-tandem mass spectrometry (LC-MS/MS) and identified with authentic GPP, (*E*,*E*)-FPP, and (*E*,*E*,*E*)-GGPP. **(B)** For testing the influence of the metal ion cofactor on product specificity, purified ApIDS was incubated with IPP, DMAPP, and 1 mM of cofactor, and product formation was analyzed using LC-MS/MS. Quantification of enzyme products was performed using standard curves made from authentic GPP, (*E*,*E*)-FPP, and (*E*,*E*,*E*)-GGPP. Means and SE are shown (n = 3 technical replicates).

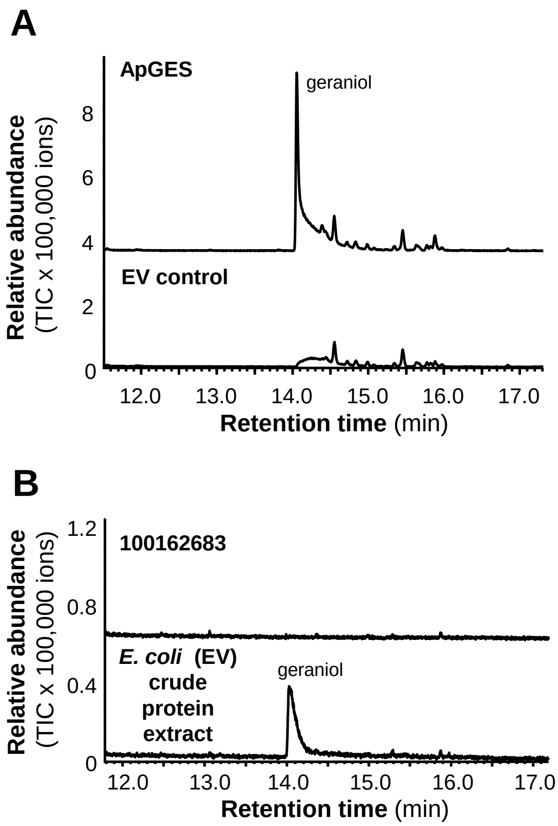

Supplemental Figure S3: ApGES showed phosphatase activity with GPP. (A) The phosphatase ApGES was expressed in *Saccharomyces cerevisiae* and microsomes harboring the recombinant protein were incubated with GPP. Geraniol was extracted with hexane and analyzed using gas chromatography-mass spectrometry (GC-MS). Microsomes prepared from *S. cerevisiae* carrying the empty expression vector were used as negative control. **(B)** Another potential phosphatase candidate (gene ID 100162683) annotated as inositol polyphosphate 1-phosphatase was expressed in *Escherichia coli,* purified, and incubated with GPP. A crude protein extract made from *E. coli* carrying the empty expression vector and fed with GPP showed unspecific phosphatase activity and was used as positive control. Reaction products were extracted with hexane and analyzed using GC-MS.

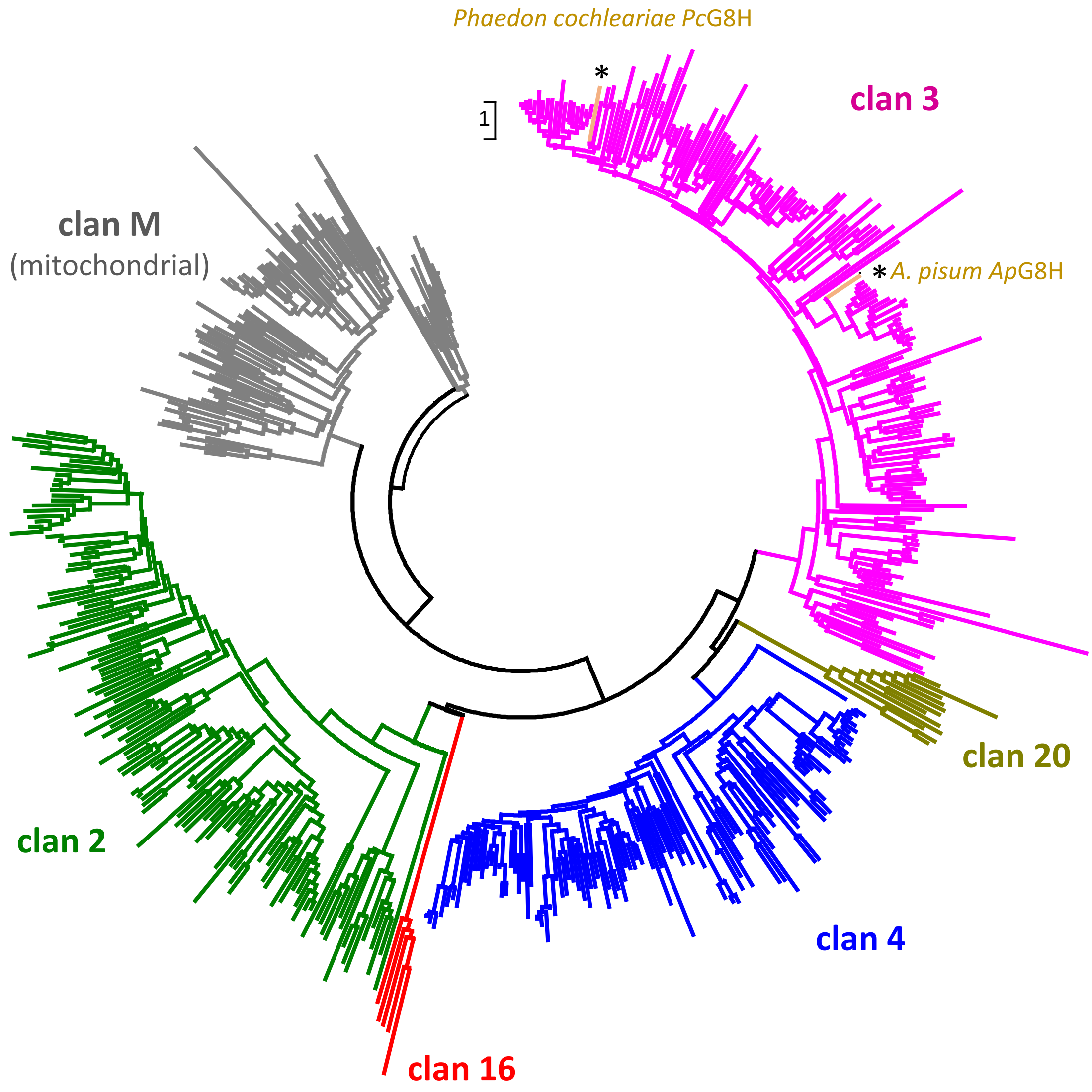

Supplemental Figure S4: ApG8H from *A. pisum* and PcG8H from *P. cochleariae* both belong to the clan 3 of Arthropode P450s, but share only 35% amino acid similarity. Cladogram analysis of Arthropode P450 proteins including ApG8H from *A. pisum* and PcG8H from *P. cochleariae*. The tree was inferred by using the Neighbor-Joining method based on the JTT matrix-based method. The rate variation among sites was modeled with a gamma distribution (shape parameter = 1). The analysis involved 542 amino acid sequences. All positions with less than 80% site coverage were eliminated. The tree is drawn to scale, with branch length measured in the number of amino acid substitutions per site.

A

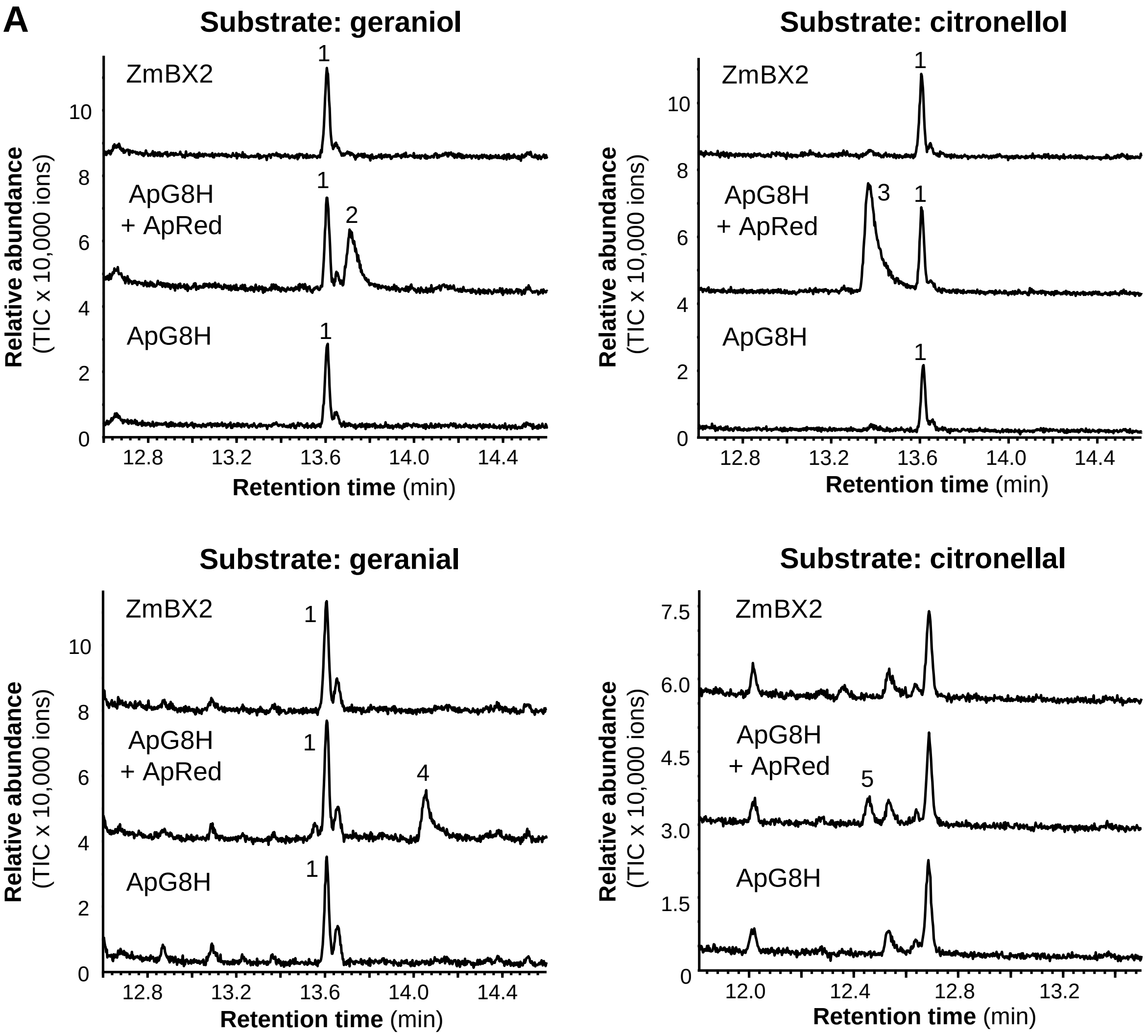

B

	ApG8H	ApG8H + ApRed
geraniol	-	+
citral A+B	-	+
nerol	-	+
citronellol	-	+
linalool	-	+
limonene	-	-
myrcene	-	-

Supplemental Figure S5: Biochemical characterization of ApG8H. (A) Yeast (*Saccharomyces cerevisiae*) microsomes containing either ApG8H, ApG8H in combination with the P450 reductase ApRed, or maize ZmBX2 as negative control were assayed with potential terpenoid substrates and NADPH as cosubstrate. Reaction products were analyzed using GC-MS. 1, di-tert-butylphenol (contamination); 2, 8-hydroxygeraniol; 3, 8-hydroxycitronellol; 4, 8-hydroxygeranial; 5, 8-hydroxycitronellal. Compounds were identified by comparisons with authentic standards (8-hydroxygeraniol) or the NIST, WILEY, and Adams mass spec libraries. **(B)** *S. cerevisiae* liquid cultures expressing either *ApG8H* alone or in combination with *ApRed* were fed with potential terpenoid substrates. Reaction products were extracted with ethylacetate from the cultures and analyzed using GC-MS. +, activity could be observed; -, no activity.

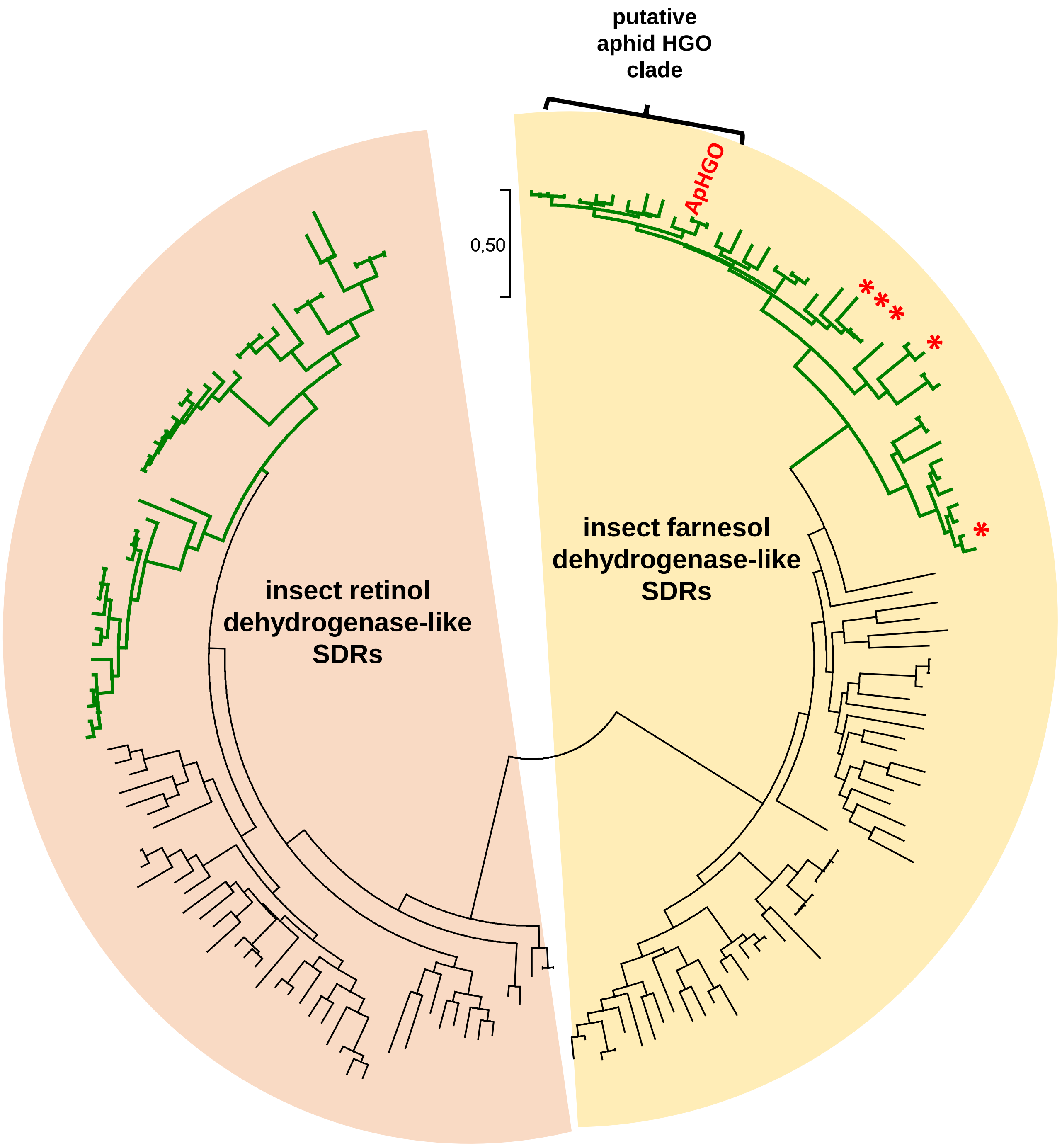

Supplemental figure S6: ApHGO belongs to the aphid clade of farnesol dehydrogenase-like short-chain dehydrogenases/reductases (SDRs). ApHGO is shown in red and other farnesol dehydrogenase-like SDRs from *A. pisum* are marked with red asterisks. The tree was inferred by using the Maximum Likelihood method based on the JTT matrix-based model and is drawn to scale, with branch lengths measured in the number of amino acid substitutions per site. Aphid proteins are marked with green branches.

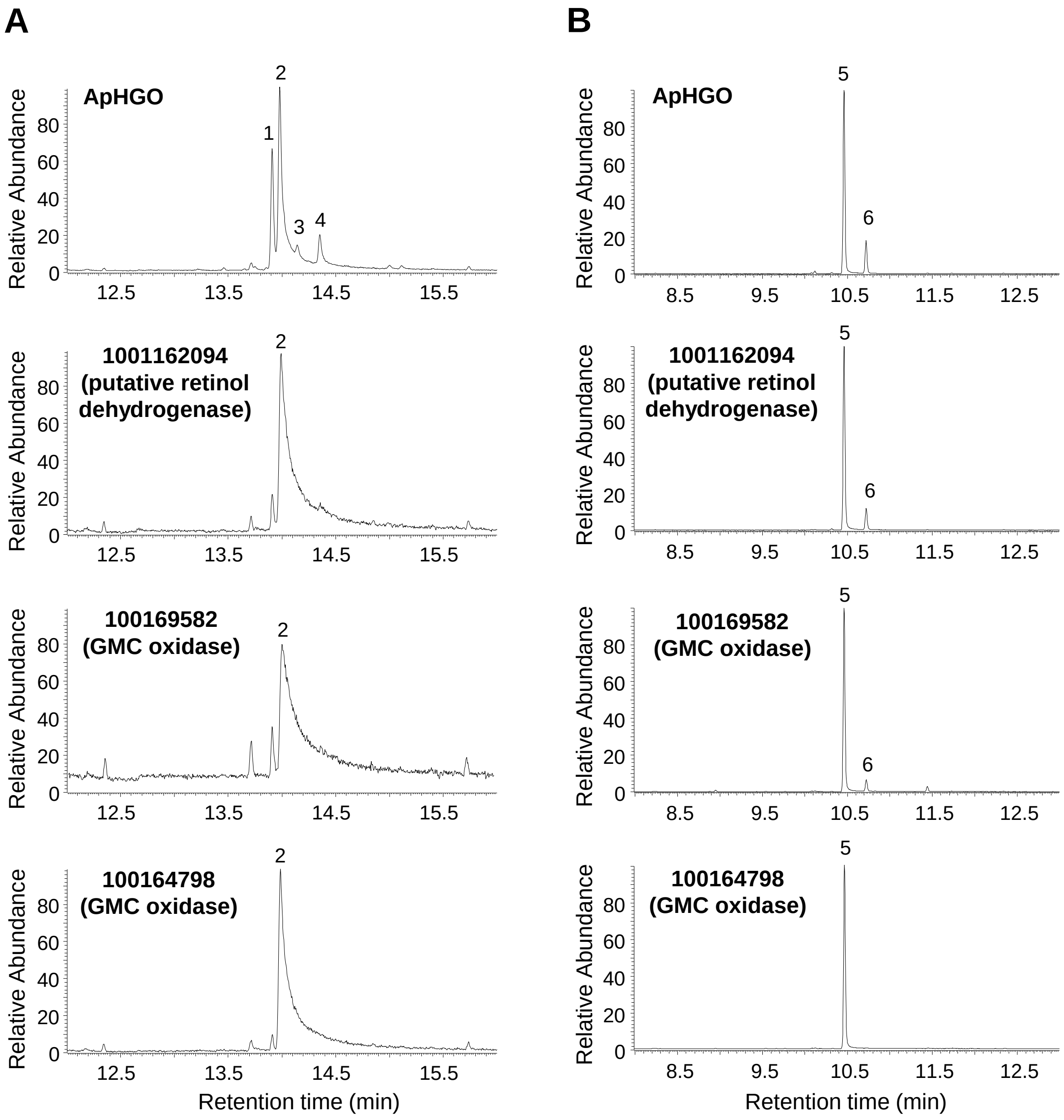

Supplemental Figure S7: The putative retinol dehydrogenase 1001162094 and the GMC oxidase 100169582 (ApNEPO) accepted geraniol but not 8-hydroxygeraniol as substrate. Enzymes were expressed as N-terminal His-tag-fusion proteins in *Escherichia coli*, purified, and incubated with 8-hydroxygeraniol **(A)** or geraniol **(B)** in the presence of NADP. Enzyme products were extracted from the assays and analyzed using gas chromatography-mass spectrometry. 1, 8-oxogeraniol; 2, 8-hydroxygeraniol; 3, 8-oxogeranial; 4, 8-hydroxygeranial; 5, geraniol; 6, geranial. The GMC oxidase 100164798 showed no activity with the tested substrates.

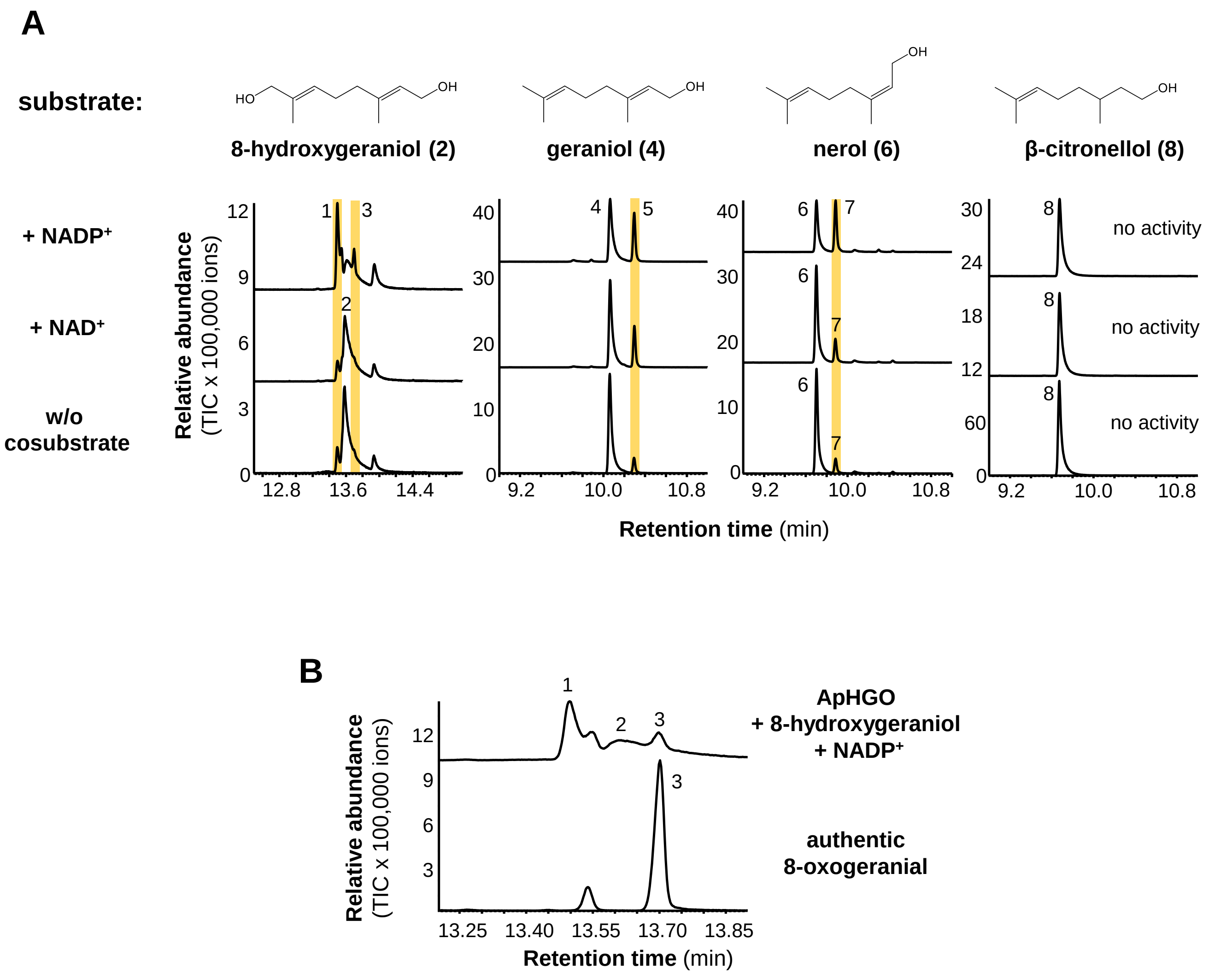

Supplemental Figure S8: Biochemical characterization of ApHGO. (A) ApHGO was expressed as N-terminal His-tag-fusion protein in *Escherichia coli*, purified, and incubated with potential terpenoid substrates either in the absence or presence of NAD(P). Enzyme products were extracted from the assays and analyzed using gas chromatography-mass spectrometry. 1, 8-oxogeraniol (partially oxidized product); 2, 8-hydroxygeraniol; 3, 8-oxogeranial (fully oxidized product); 4, geraniol; 5, geranial; 6, nerol; 7, neral; 8, β-citronellol. **(B)** The ApHGO reaction product 8-oxogeranial were identified using an authentic standard.

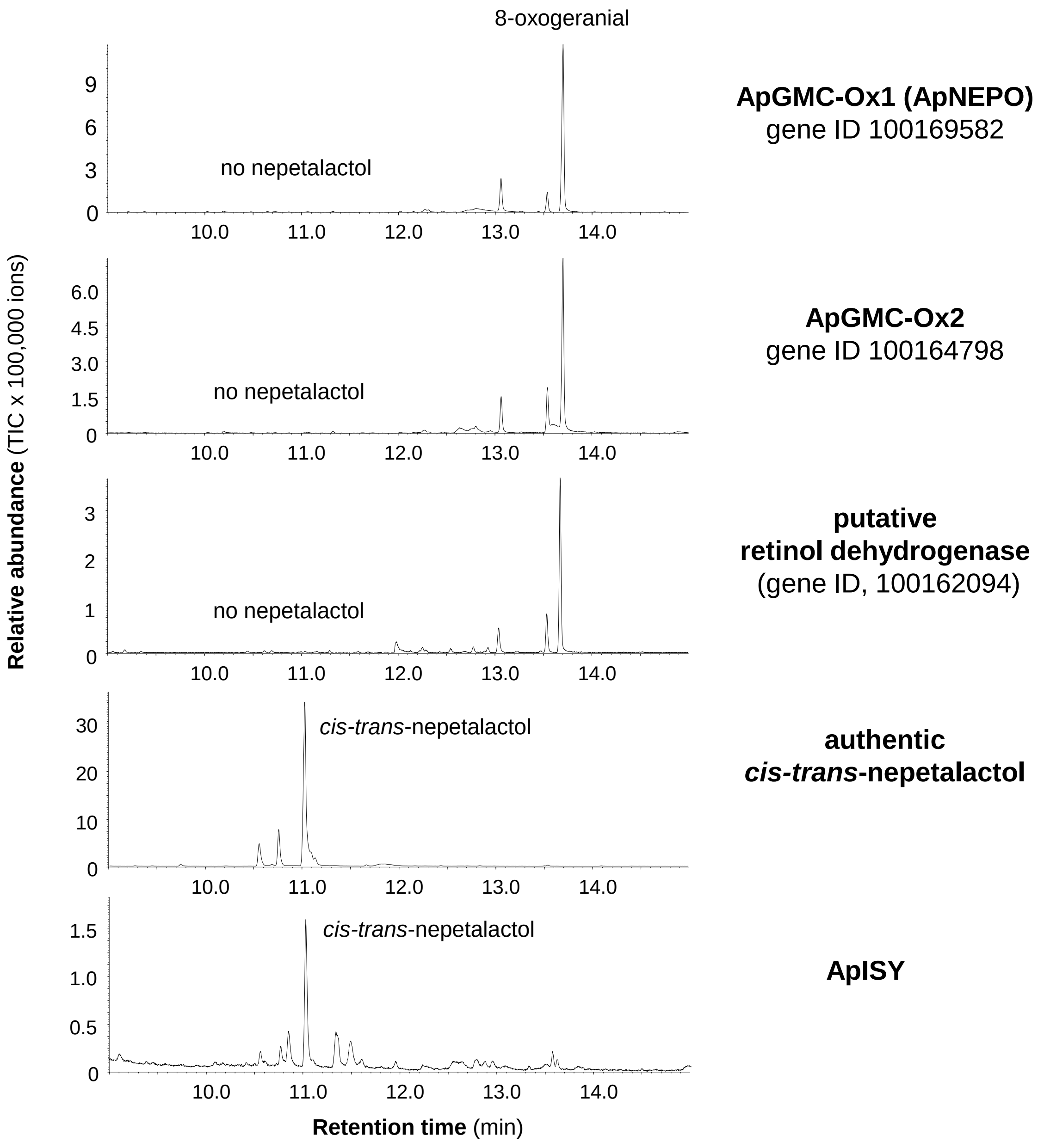

Supplemental Figure S9: Characterization of *A. pisum* oxidoreductase candidate enzymes. The two GMC oxidases and the putative retinol dehydrogenase were expressed as N-terminal His-tag-fusion proteins in *Escherichia coli*, purified, and incubated with 8-oxogeranial and NADPH. ApISY was expressed in *Saccharomyces cerevisiae* and yeast microsomes harboring the recombinant protein were fed with 8-oxogeranial and NADPH. Reaction products were extracted from the assays and analyzed using gas chromatography-mass spectrometry.

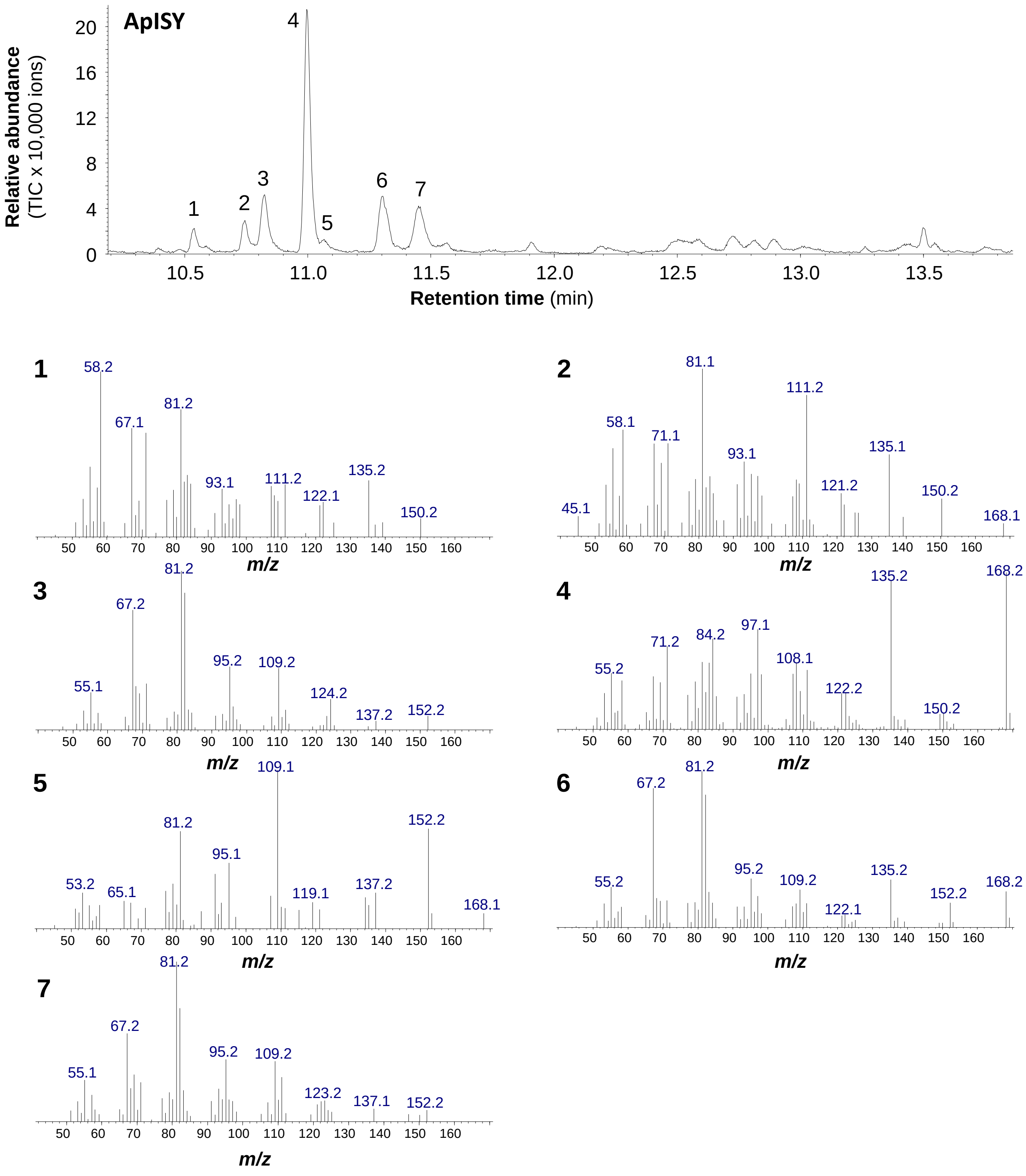

Supplemental Figure S10: Mass spectra of the ApISY reaction products. Yeast (*Saccharomyces cerevisiae*) microsomes containing ApISY were assayed with 8-oxogeranial as substrate and NADPH as cosubstrate. Reaction products were extracted with ethylacetate and analyzed using gas chromatography-mass spectrometry. 1, *cis-trans*-iridodial; 2, unidentified; 3, unidentified; 4, *cis-trans*-nepetalactol; 5, unidentified; 6, unidentified; 7, unidentified.

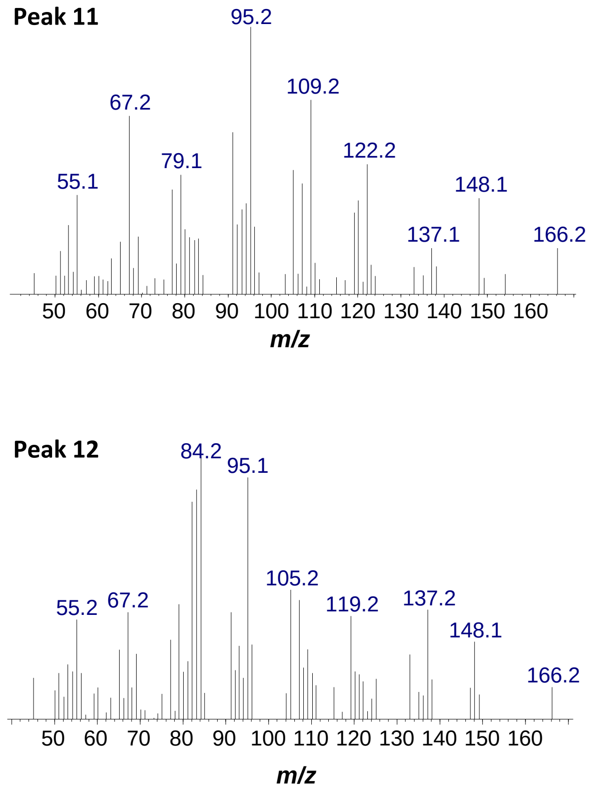

Supplemental figure S11: Mass spectra of peaks 11 and 12 in Figure 4.

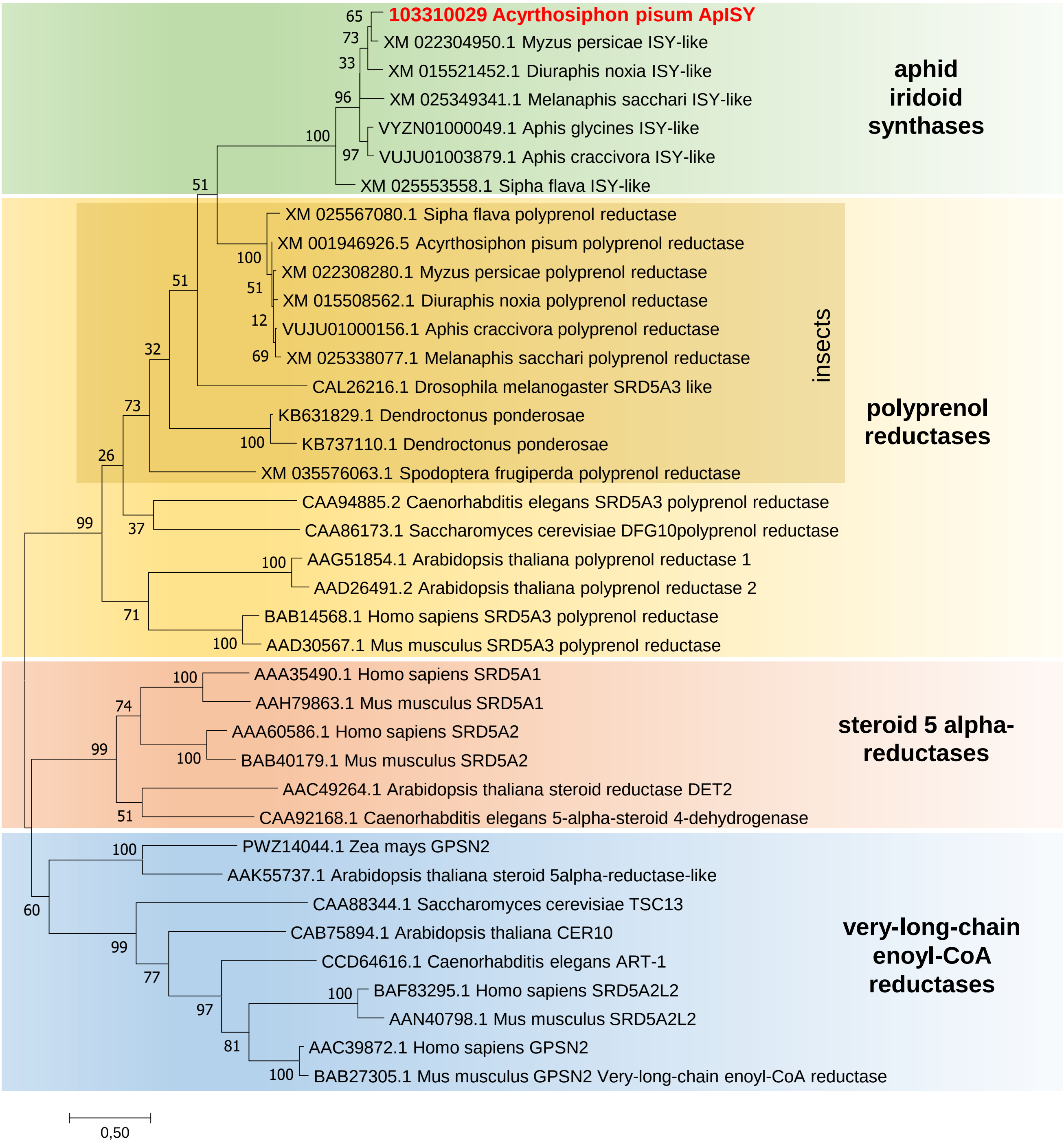

Supplemental Figure S12: ApISY belongs to the steroid-5-alpha reductase-like family (SRD5A-like). The tree was inferred by using the Maximum Likelihood method based on the JTT matrix-based model and is drawn to scale, with branch lengths measured in the number of amino acid substitutions per site. All positions with less than 80% site coverage were eliminated. Bootstrap values are given next to each node (n = 1000).

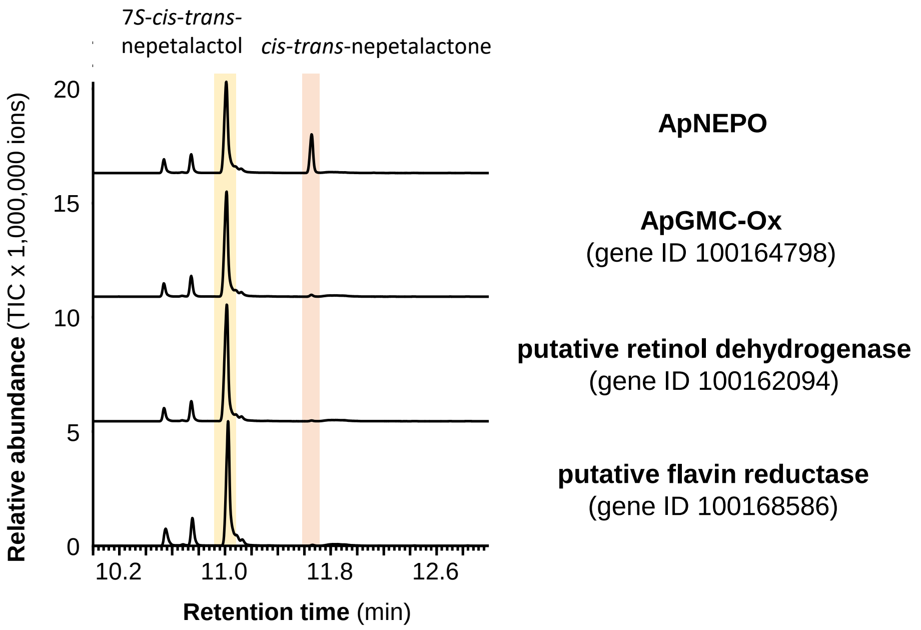

Supplemental Figure S13: Biochemical characterization of ApNEPO. ApNEPO and three other putative oxidoreductases specifically expressed in hind legs of female *A. pisum* were expressed as N-terminal His-tag-fusion proteins in *Escherichia coli*, purified, and incubated with 7*S*-*cis*-*trans*-nepetalactol in the presence of NADP. Enzyme products were extracted from the assays with ethylacetate and analyzed using gas chromatography-mass spectrometry.

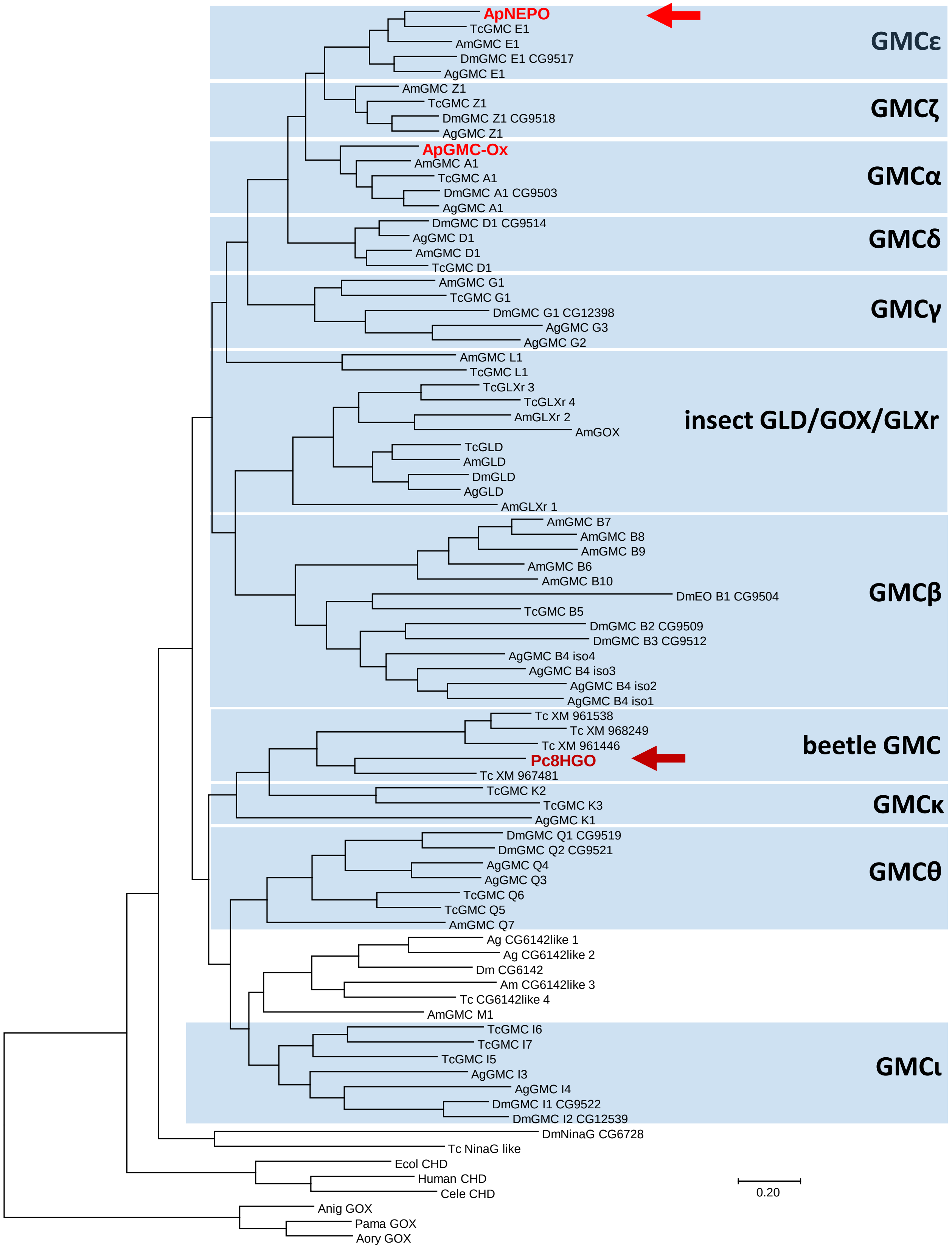

Supplemental figure S14: ApNEPO belongs to the GMCε clade of GMC oxidases and is not related to Pc8HGO from *Phaedon cochleariae*. The tree was inferred by using the Maximum Likelihood method based on the JTT matrix-based model and is drawn to scale, with branch lengths measured in the number of amino acid substitutions per site.

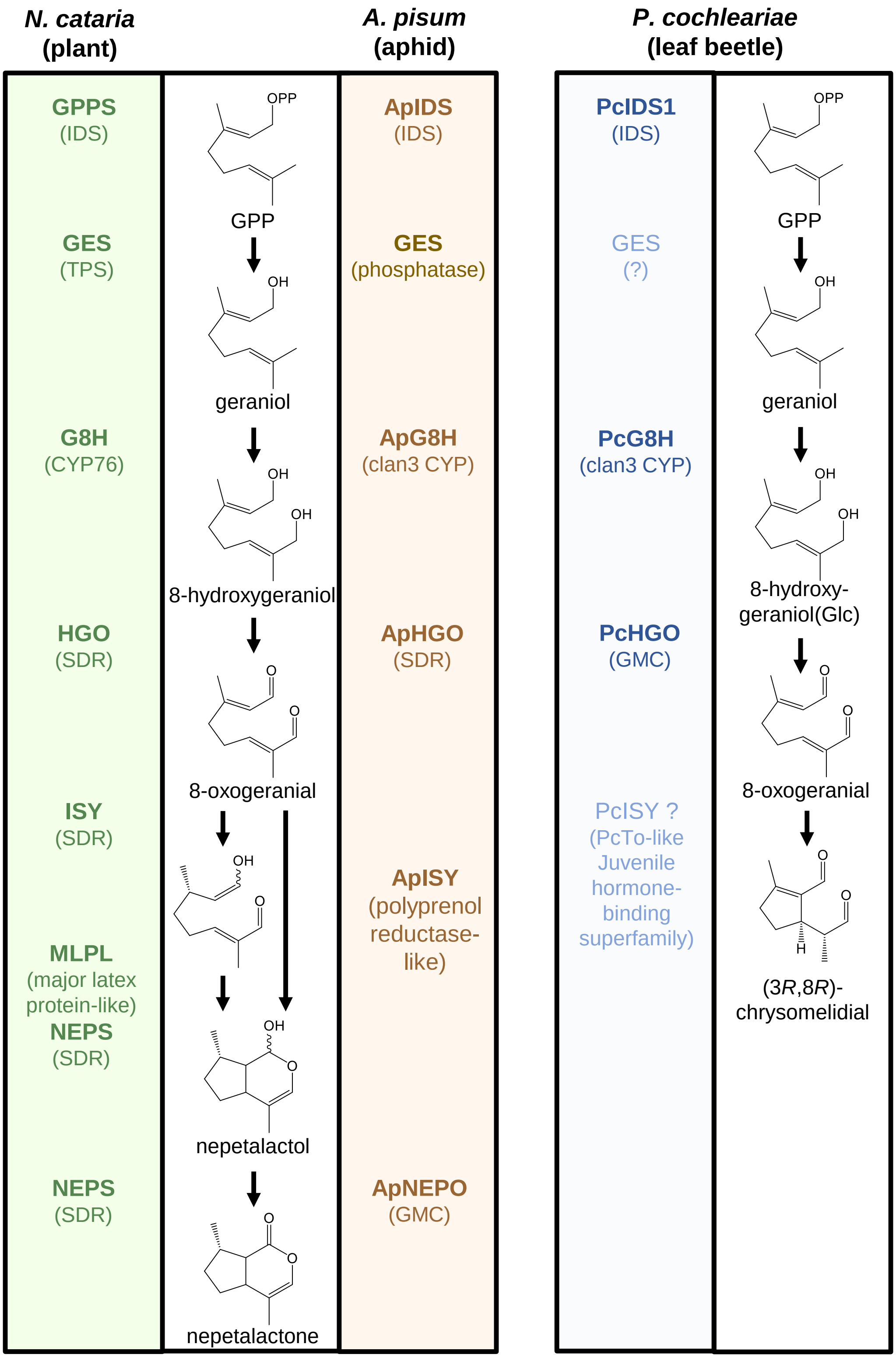

Supplemental Figure S15: The iridoid pathways in plants, aphids, and beetles evolved independently from each other. While the plant and aphid pathways both lead to nepetalactol, the beetle *Phaedon cochleariae* produces chrysomelidial. Enzymes identified and characterized are shown in bold. IDS, isoprenyldiphosphate synthase; TPS, terpene synthase; CYP, cytochrome P450 monooxygenase; SDR, short-chain dehydrogenase/reductase; GMC, glucose-methanol-cholin oxidoreductase.

Table S1: Genes selectively expressed in hind legs of sexual female pea aphids.

Parameters: FPKM hind legs >= 10; Pearson >= 0.99; FC >= 5

	gene_id	gene_description
	100164798	glucose dehydrogenase [FAD%2C quinone]%2C transcript variant X2 && P18173.3 RecName: Full=Glucose dehydrogenase [
	100165154	hydroxymethylglutaryl-CoA synthase 1%2C transcript variant X1 && P54961.1 RecName: Full=Hydroxymethylglutaryl-CoA s
ApISY	103310029	polyprenol reductase-like && Q9VLP9.1 RecName: Full=Polyprenol reductase
	100166804	uncharacterized LOC100166804 && -
	100165462	3-hydroxy-3-methylglutaryl-coenzyme A reductase && P54960.1 RecName: Full=3-hydroxy-3-methylglutaryl-coenzyme A re
ApNEPO	100169582	glucose dehydrogenase [FAD%2C quinone] && P18173.3 RecName: Full=Glucose dehydrogenase [FAD, quinone]; Contains:
	100158949	mitochondrial 2-oxoglutarate/malate carrier protein-like%2C transcript variant X3 && Q9CR62.3 RecName: Full=Mitochond
	103309505	4-coumarate--CoA ligase-like 7 && Q26304.1 RecName: Full=Luciferin 4-monooxygenase; Short=Luciferase
	100162815	acetyl-CoA acetyltransferase%2C cytosolic%2C transcript variant X2 && Q9BWD1.2 RecName: Full=Acetyl-CoA acetyltransfe
	107883989	uncharacterized LOC107883989 && -
	100161127	protein dispatched && Q96F81.3 RecName: Full=Protein dispatched homolog 1
	100158798	diphosphomevalonate decarboxylase%2C transcript variant X3 && Q5U403.1 RecName: Full=Diphosphomevalonate decarb
	100159664	mitochondrial 2-oxoglutarate/malate carrier protein && Q9CR62.3 RecName: Full=Mitochondrial 2-oxoglutarate/malate ca
	100163938	EF-hand calcium-binding domain-containing protein 4A%2C transcript variant X2 && A1A600.2 RecName: Full=EF-hand calc
	107882164	uncharacterized LOC107882164 && -
	100162573	androgen-induced gene 1 protein && Q9D8B1.1 RecName: Full=Androgen-induced gene 1 protein; Short=AIG-1
	103311408	gustatory and odorant receptor 22-like && -
	100572104	uncharacterized LOC100572104 && -
	100572448	uncharacterized LOC100572448%2C transcript variant X2 && -
	100168586	biliverdin reductase B (flavin reductase (NADPH)) && P30043.3 RecName: Full=Flavin reductase (NADPH); Short=FR; AltNam
	100161879	DEP domain-containing protein 1A%2C transcript variant X3 && Q803Q4.1 RecName: Full=DEP domain-containing protein 1
	100166744	isopentenyl diphosphate isomerase && Q4R4W5.2 RecName: Full=Isopentenyl-diphosphate Delta-isomerase 1; AltName: F
	100165234	putative transcription factor SOX-15 && P40657.2 RecName: Full=Putative transcription factor SOX-15; AltName: Full=Sox5
	100162856	uncharacterized LOC100162856 && -
	100163413	phosphomevalonate kinase-like && Q29081.4 RecName: Full=Phosphomevalonate kinase; Short=PMKase
	100165274	graves disease carrier protein && Q8C0K5.1 RecName: Full=Graves disease carrier protein homolog; Short=GDC; AltName: I
ApG8H	100165972	probable cytochrome P450 6a13 && Q9V4U9.1 RecName: Full=Probable cytochrome P450 6a13; AltName: Full=CYPVIA13
	100159735	cuticular protein 54 && P11734.1 RecName: Full=Cuticle protein 8; AltName: Full=LM-ACP 8; Short=LM-8
	100162683	inositol polyphosphate 1-phosphatase%2C transcript variant X2 && P49442.2 RecName: Full=Inositol polyphosphate 1-pho
	100166560	4-coumarate--CoA ligase-like 9 && Q26304.1 RecName: Full=Luciferin 4-monooxygenase; Short=Luciferase
	100574505	phosphomevalonate kinase && Q29081.4 RecName: Full=Phosphomevalonate kinase; Short=PMKase
	100162664	tumor necrosis factor alpha-induced protein 8-like protein%2C transcript variant X3 && Q28ZG0.1 RecName: Full=Tumor ne
	100574136	flavin reductase (NADPH) && P30043.3 RecName: Full=Flavin reductase (NADPH); Short=FR; AltName: Full=Biliverdin reduc
ApIDS	100144905	mitochondrial isoprenyl diphosphate synthase && P08836.2 RecName: Full=Farnesyl pyrophosphate synthase; Short=FPP s
	100169498	mRNA-capping enzyme%2C transcript variant X2 && Q6NY98.1 RecName: Full=mRNA-capping enzyme; Includes: RecName
	100160431	uncharacterized LOC100160431%2C transcript variant X2 && -
	100160076	armadillo repeat-containing protein gudu-like && B2RY50.1 RecName: Full=Armadillo repeat-containing protein 4
	100169041	uncharacterized LOC100169041 && Q6X0I2.1 RecName: Full=Vitellogenin receptor; Short=SiVgR; Flags: Precursor
	100162264	RUN domain-containing protein 1 && Q0VDN7.1 RecName: Full=RUN domain-containing protein 1
	103307655	ABC transporter G family member 23 && Q55EH8.2 RecName: Full=ABC transporter G family member 23; AltName: Full=AE
	100161003	MICAL-like protein 2%2C transcript variant X2 && Q8N3F8.2 RecName: Full=MICAL-like protein 1; AltName: Full=Molecule i
	103311672	protein 5NUC-like && Q9XZ43.1 RecName: Full=Protein 5NUC; Includes: RecName: Full=UDP-sugar hydrolase; AltName: Ful
	100161212	protein 5NUC && Q9XZ43.1 RecName: Full=Protein 5NUC; Includes: RecName: Full=UDP-sugar hydrolase; AltName: Full=UI
	100159865	katanin p60 ATPase-containing subunit A-like 1%2C transcript variant X3 && Q5U3S1.1 RecName: Full=Katanin p60 ATPase-
	100159902	neural proliferation differentiation and control protein 1%2C transcript variant X1 && Q64322.2 RecName: Full=Neural prol
	100169605	lysophosphatidylcholine acyltransferase && Q0KHU5.1 RecName: Full=Lysophosphatidylcholine acyltransferase; AltName: F
	115034333	uncharacterized LOC115034333 && -
	100162731	UDP-glucuronosyltransferase 1-3%2C transcript variant X2 && O77649.1 RecName: Full=UDP-glucuronosyltransferase 2B20
	100575510	armadillo repeat-containing protein gudu-like%2C transcript variant X2 && Q5T2S8.1 RecName: Full=Armadillo repeat-cont
	100163305	mevalonate kinase && Q86AG7.1 RecName: Full=Mevalonate kinase; Short=MK
	100569602	uncharacterized LOC100569602%2C transcript variant X1 && -
ApPhos	100158803	dolichyldiphosphatase 1%2C transcript variant X3 && B2KI79.1 RecName: Full=Dolichyldiphosphatase 1; AltName: Full=Doli
	100163040	SRSF protein kinase 3%2C transcript variant X2 && Q03563.3 RecName: Full=Serine/threonine-protein kinase spk-1
	100158932	solute carrier family 25 member 44 && Q96H78.1 RecName: Full=Solute carrier family 25 member 44
	100159558	UDP-glucuronosyltransferase 2B2-like && P54855.3 RecName: Full=UDP-glucuronosyltransferase 2B15; Short=UDPGT 2B15
	100164200	ras-responsive element-binding protein 1%2C transcript variant X2 && Q3UHF7.1 RecName: Full=Transcription factor HIVEI
	100571500	elongation of very long chain fatty acids protein 4%2C transcript variant X1 && Q9GZR5.1 RecName: Full=Elongation of very
	100160352	two pore potassium channel protein sup-9%2C transcript variant X2 && O17185.2 RecName: Full=Two pore potassium char
	100569853	uncharacterized LOC100569853 && -
	100166089	venom carboxylesterase-6 && B2D0J5.1 RecName: Full=Venom carboxylesterase-6; AltName: Allergen=Api m 8; Flags: Prec
	100165508	7-methylguanosine phosphate-specific 5'-nucleotidase && Q969T7.4 RecName: Full=7-methylguanosine phosphate-specific
	100160499	F-box only protein 32 && Q4R372.1 RecName: Full=F-box only protein 25
	100161393	probable multidrug resistance-associated protein lethal(2)03659 && P91660.4 RecName: Full=Probable multidrug resistanc
	100169450	ELOVL family member 6%2C elongation of long chain fatty acids && Q5ZJR8.1 RecName: Full=Elongation of very long chain

	100162877	uncharacterized LOC100162877%2C transcript variant X1 && -
	100574123	uncharacterized LOC100574123 && -
	100168638	ATP-binding cassette sub-family G member 1 && P45844.3 RecName: Full=ATP-binding cassette sub-family G member 1; Al[illegible]
	100163010	NADP-dependent malic enzyme && P28227.1 RecName: Full=NADP-dependent malic enzyme; Short=NADP-ME
	100159357	1%2C5-anhydro-D-fructose reductase && O08782.3 RecName: Full=Aldose reductase-related protein 2; Short=AR; AltName[illegible]
	100162924	NACHT and WD repeat domain-containing protein 2 && Q9ULI1.3 RecName: Full=NACHT and WD repeat domain-containin[illegible]
	100574710	pancreatic triacylglycerol lipase && Q17RR3.2 RecName: Full=Pancreatic lipase-related protein 3; Short=PL-RP3; Flags: Prec[illegible]
	100168408	protein SERAC1%2C transcript variant X1 && Q2TBM9.1 RecName: Full=Protein SERAC1; AltName: Full=Serine active site-c[illegible]
	100165720	NAD kinase%2C transcript variant X3 && P58058.2 RecName: Full=NAD kinase; AltName: Full=Poly(P)/ATP NAD kinase
	100167523	serine/threonine-protein kinase SIK1%2C transcript variant X1 && Q9H0K1.1 RecName: Full=Serine/threonine-protein kinas[illegible]
	100163144	putative inorganic phosphate cotransporter%2C transcript variant X3 && Q9V7S5.1 RecName: Full=Putative inorganic phos[illegible]
	100167870	lysophospholipid acyltransferase 1%2C transcript variant X2 && Q8BH98.1 RecName: Full=Lysophospholipid acyltransferase[illegible]
	100570401	fructose-1%2C6-bisphosphatase 1%2C transcript variant X2 && P00636.4 RecName: Full=Fructose-1,6-bisphosphatase 1; Sh[illegible]
	100161475	fructose-1%2C6-bisphosphatase 1 && P00637.4 RecName: Full=Fructose-1,6-bisphosphatase 1; Short=FBPase 1; AltName: [illegible]
	100571243	gamma-glutamyl hydrolase A-like && Q54LN4.1 RecName: Full=Gamma-glutamyl hydrolase A; AltName: Full=Conjugase A;
	100169524	hexokinase type 2%2C transcript variant X2 && Q9NFT7.4 RecName: Full=Hexokinase type 2
**ApHGO**	100301633	short-chain dehydrogenase/reductase-like && D2WKD9.2 RecName: Full=Farnesol dehydrogenase; AltName: Full=NADP+-[illegible]
	100160720	UDP-glucose pyrophosphorylase 2-like%2C transcript variant 1 && Q91ZJ5.3 RecName: Full=UTP--glucose-1-phosphate urid[illegible]
	100168697	transcription initiation factor IIA subunit 1 && P52654.2 RecName: Full=Transcription initiation factor IIA subunit 1; AltNam[illegible]
**ApRED**	100160284	NADPH--cytochrome P450 reductase && Q07994.1 RecName: Full=NADPH--cytochrome P450 reductase; Short=CPR; Short[illegible]
	100159885	suppressor of hairless protein-like && P28159.1 RecName: Full=Suppressor of hairless protein; AltName: Full=J kappa-recon[illegible]
	100568673	uncharacterized LOC100568673 && -
	100165099	ATP citrate lyase && Q2TCH3.1 RecName: Full=ATP-citrate synthase; AltName: Full=ATP-citrate (pro-S-)-lyase; AltName: Ful[illegible]
	100162094	retinol dehydrogenase 11%2C transcript variant X1 && Q8TC12.2 RecName: Full=Retinol dehydrogenase 11; AltName: Full=[illegible]
	100159025	uncharacterized LOC100159025%2C transcript variant X5 && -
	100160153	glycerol-3-phosphate dehydrogenase%2C mitochondrial%2C transcript variant X2 && Q64521.2 RecName: Full=Glycerol-3-[illegible]
	100162951	organic cation transporter protein && O76082.1 RecName: Full=Solute carrier family 22 member 5; AltName: Full=High-affi[illegible]
	100167416	uncharacterized LOC100167416 && -
	100167746	heat shock protein 67B2-like%2C transcript variant X1 && Q9D0B5.1 RecName: Full=Thiosulfate sulfurtransferase/rhodanes[illegible]
	100160658	glycerol-3-phosphate dehydrogenase [NAD(+)]%2C cytoplasmic%2C transcript variant X2 && O97463.3 RecName: Full=Glyc[illegible]
	100163328	uncharacterized LOC100163328%2C transcript variant X1 && Q5F4B8.1 RecName: Full=Solute carrier family 46 member 3; [illegible]
	100571790	serine palmitoyltransferase 2 && P97363.2 RecName: Full=Serine palmitoyltransferase 2; AltName: Full=Long chain base bi[illegible]

mevalonate pathway genes

potential iridoid gene candidates

potential transporters for iridoid precursors

average RPKM						RPKM	
100	1	1	1			female-hind 1	female-hind 2
**female-hind**	**female-front**	**parth-hind**	**male-hind**	**Pearson**	**average FC**	f_hi_H5	f_hi_FH8
3382.7	0.0	0.1	0.7	1.00000	12182.3	3632.3	3072.2
5143.0	3.7	4.5	5.1	1.00000	1164.6	5241.9	4922.5
140.6	0.0	0.0	0.1	1.00000	5125.5	137.3	137.1
980.0	0.9	0.1	0.0	1.00000	2930.2	998.8	1023.7
1502.1	18.6	16.9	17.9	1.00000	84.4	1545.3	1463.5
28.5	0.0	0.0	0.0	1.00000	2054.6	29.1	27.7
148.1	0.4	0.2	0.2	1.00000	598.0	157.2	131.0
117.6	0.3	0.1	0.3	1.00000	542.9	114.5	121.1
1298.5	9.6	11.6	8.3	1.00000	132.0	1234.1	1270.4
147.2	0.4	0.7	0.4	1.00000	297.9	149.1	135.4
34.2	1.0	1.0	1.1	1.00000	33.3	34.7	33.3
2094.0	29.5	36.3	31.6	1.00000	64.5	2100.3	2011.0
640.2	60.4	61.3	58.7	0.99999	10.6	648.0	629.9
117.3	0.7	1.0	1.3	0.99999	117.8	119.7	112.7
65.0	0.7	0.4	0.5	0.99999	120.5	71.5	60.6
2712.7	14.0	0.7	1.9	0.99999	489.4	2971.6	2423.2
63.7	0.5	0.1	0.2	0.99999	223.4	65.1	63.1
24.7	0.7	0.5	0.6	0.99999	42.1	26.9	21.4
84.9	0.5	0.1	0.6	0.99999	216.4	96.2	70.7
266.9	31.8	33.2	32.2	0.99999	8.2	251.7	266.9
131.8	1.8	1.7	2.5	0.99999	66.8	137.0	123.3
2136.3	44.5	29.2	34.5	0.99998	59.3	2200.0	2005.1
90.6	3.2	3.9	3.8	0.99997	24.9	96.0	80.0
273.8	4.1	6.4	4.9	0.99997	53.4	286.2	255.3
231.0	4.3	4.7	6.3	0.99997	45.5	237.9	214.3
737.4	21.8	15.5	21.4	0.99997	37.7	797.0	679.9
3409.3	13.2	24.5	48.4	0.99996	118.8	3307.5	3455.9
51.7	0.1	0.2	0.6	0.99996	179.3	48.3	51.4
19.2	0.9	0.8	1.0	0.99996	21.7	23.3	15.8
10.1	0.1	0.0	0.1	0.99996	162.3	10.1	10.8
1447.1	21.9	26.3	39.4	0.99995	49.6	1463.3	1396.2
36.0	2.5	2.4	3.0	0.99988	13.6	42.8	30.5
299.5	37.0	32.2	32.9	0.99987	8.8	294.1	281.3
395.8	20.3	13.3	13.9	0.99986	25.0	396.8	391.1
48.5	6.9	7.7	7.1	0.99984	6.7	48.2	44.4
185.6	2.9	6.7	5.9	0.99983	35.9	198.4	173.1
99.9	1.4	1.4	3.4	0.99982	48.9	130.2	80.1
67.7	1.0	2.3	0.9	0.99980	48.9	71.5	68.2
39.3	2.1	3.0	2.3	0.99979	15.9	41.8	36.9
17.1	0.3	0.6	0.7	0.99979	31.4	21.6	14.4
110.5	9.3	10.6	8.1	0.99979	11.8	115.0	100.8
80.7	12.1	10.6	10.6	0.99978	7.3	86.0	76.3
478.6	70.4	62.9	59.2	0.99975	7.5	524.2	435.8
48.7	8.6	7.9	7.4	0.99971	6.1	51.2	44.0
77.9	6.0	4.6	6.7	0.99970	13.5	79.8	70.6
178.9	32.4	28.0	31.0	0.99969	5.9	174.1	172.5
141.2	14.8	11.5	15.1	0.99968	10.2	142.8	134.1
259.0	2.8	10.6	5.1	0.99966	41.9	277.1	236.2
69.7	2.3	3.4	4.6	0.99960	20.4	87.0	58.9
374.4	37.3	28.5	25.6	0.99959	12.3	376.3	366.0
12.3	0.5	0.3	0.8	0.99956	22.5	12.1	12.1
122.3	5.9	10.2	8.5	0.99952	14.9	130.7	113.2
36.8	1.7	3.2	2.3	0.99934	15.3	39.4	33.8
78.2	6.9	9.9	7.7	0.99934	9.6	80.7	70.0
11.0	0.1	0.1	0.5	0.99933	51.2	9.1	11.2
40.4	4.7	3.0	3.9	0.99926	10.5	42.0	36.2
10.7	0.4	0.7	0.2	0.99916	25.4	12.2	9.9
31.1	1.2	2.6	1.4	0.99912	17.9	32.2	27.6
24.2	0.4	1.5	0.6	0.99910	28.4	22.5	24.3
876.4	119.1	123.5	154.8	0.99909	6.6	964.0	839.0
156.6	15.1	7.5	10.0	0.99905	14.4	145.6	160.3
157.8	26.6	25.9	32.7	0.99888	5.6	170.6	154.7
306.4	41.6	25.7	31.0	0.99883	9.3	305.0	299.6
1180.7	80.2	29.2	15.4	0.99881	28.4	1235.3	1105.1

25.7	0.5	1.8	0.6	0.99879	26.5	25.2	24.5
75.1	0.2	4.6	2.0	0.99876	33.7	89.4	63.4
96.1	15.8	10.5	11.5	0.99849	7.6	100.4	88.7
3475.4	334.8	122.5	157.9	0.99839	16.9	3539.5	3409.6
5558.8	113.0	461.5	407.9	0.99829	17.0	5795.8	5301.3
23.7	2.0	2.6	3.5	0.99829	8.8	24.4	21.4
38.0	2.4	0.2	0.0	0.99824	43.4	42.7	32.2
33.1	6.3	4.5	4.5	0.99822	6.5	36.2	32.8
236.5	31.0	41.7	44.8	0.99821	6.0	214.1	220.5
128.2	6.3	15.7	10.8	0.99789	11.7	131.1	120.8
98.7	11.8	16.3	18.9	0.99754	6.3	101.9	92.2
182.3	32.7	19.3	20.6	0.99709	7.5	194.2	173.7
1013.0	126.9	101.9	182.4	0.99706	7.4	1064.9	953.6
367.0	46.1	35.3	65.5	0.99693	7.5	387.2	354.9
38.3	4.0	0.6	1.2	0.99655	19.9	42.3	34.8
2036.8	412.3	452.4	283.4	0.99622	5.3	2036.0	1981.2
10416.5	219.4	1062.1	1207.2	0.99590	12.6	10529.8	9669.1
921.8	219.3	131.8	164.3	0.99540	5.4	864.9	942.8
223.4	47.4	28.0	27.9	0.99536	6.5	222.2	220.0
804.9	91.7	159.0	162.2	0.99528	5.8	812.8	780.4
49.4	5.0	6.2	10.3	0.99417	6.9	59.2	46.8
79.7	2.3	11.3	2.6	0.99371	14.7	79.2	79.7
1601.9	302.6	245.1	107.0	0.99304	7.3	1598.9	1562.4
96.6	12.4	0.5	0.5	0.99272	21.7	93.6	95.5
94.8	12.5	17.8	5.0	0.99214	8.1	104.6	88.2
908.4	133.2	244.8	166.1	0.99180	5.0	907.5	865.5
82.6	6.2	16.9	14.3	0.99171	6.6	84.5	78.1
505.1	111.4	72.4	43.0	0.99159	6.7	436.6	485.3
10.1	1.2	2.5	2.0	0.99138	5.3	11.9	8.7
841.9	103.1	216.4	153.3	0.99095	5.3	858.0	803.6
51.5	8.9	2.2	1.5	0.99021	12.3	54.9	48.2
16.7	3.5	1.2	1.3	0.99006	8.3	16.2	16.5

female-hind 3	female-front 1	female-front 2	female-front 3	parth-hind 1	parth-hind 2	parth-hind 3	male-hind 1	male-hind 2
f_hi_FH9	f_fr_V1	f_fr_V2	f_fr_V3	p_hi_PH2	p_hi_PH4	p_hi_PH5	m_hi_MH1	m_hi_MH3
3443.5	0.0	0.1	0.0	0.3	0.0	0.0	0.2	1.6
5264.7	3.3	4.2	3.7	4.4	4.4	4.6	5.1	5.2
147.4	0.0	0.0	0.0	0.0	0.0	0.0	0.0	0.1
917.4	0.8	1.2	0.8	0.1	0.1	0.0	0.0	0.0
1497.6	19.3	19.3	17.4	15.9	17.1	17.6	17.1	17.5
28.8	0.0	0.0	0.0	0.0	0.0	0.0	0.1	0.0
155.9	0.4	0.3	0.3	0.2	0.3	0.1	0.1	0.3
117.2	0.3	0.2	0.5	0.2	0.0	0.0	0.3	0.5
1391.1	10.1	10.3	8.5	12.7	11.2	10.9	7.9	9.0
156.9	0.6	0.4	0.2	0.6	0.7	0.8	0.3	0.3
34.7	0.6	0.9	1.5	0.9	1.2	0.9	1.3	1.1
2170.6	29.4	28.9	30.3	38.6	35.8	34.3	31.9	29.3
642.7	67.3	59.9	53.9	58.4	63.0	62.5	61.5	55.4
119.6	0.8	0.6	0.7	0.6	1.1	1.3	0.6	2.1
63.0	0.8	0.7	0.7	0.3	0.4	0.4	0.6	0.4
2743.3	20.9	11.3	9.8	1.0	0.7	0.4	2.7	1.9
62.8	0.5	0.3	0.7	0.1	0.3	0.0	0.3	0.1
25.9	0.6	0.8	0.6	0.6	0.5	0.5	0.4	0.6
87.7	0.3	0.4	0.8	0.2	0.0	0.1	0.6	0.6
282.0	31.7	31.7	31.9	30.4	37.6	31.6	35.0	30.3
135.1	1.9	2.0	1.5	1.6	1.7	1.7	2.1	2.9
2203.8	46.4	46.2	40.8	28.7	30.2	28.5	35.8	36.0
95.9	2.7	3.3	3.6	3.2	4.7	3.9	4.0	3.7
279.7	3.9	4.3	4.0	7.5	6.4	5.4	7.2	3.9
240.6	4.2	4.0	4.6	3.8	4.8	5.3	6.9	6.7
735.2	21.6	21.6	22.2	15.3	15.2	16.1	22.2	22.1
3464.5	12.5	11.4	15.6	24.3	26.4	22.6	41.5	54.3
55.5	0.2	0.1	0.0	0.2	0.1	0.3	0.3	0.8
18.6	0.8	0.8	1.1	0.9	0.7	0.8	1.0	0.9
9.3	0.0	0.2	0.1	0.0	0.0	0.0	0.1	0.1
1481.7	19.4	21.5	24.8	23.6	29.3	26.1	40.7	41.2
34.7	2.7	1.8	3.0	3.0	2.4	1.8	4.0	2.4
323.1	39.6	38.5	32.8	27.6	35.8	33.1	38.2	30.1
399.6	21.5	22.3	16.9	13.9	13.1	12.9	15.0	12.4
52.8	7.9	6.8	6.0	8.5	7.6	7.1	6.7	7.1
185.3	2.8	2.9	3.0	4.9	7.6	7.7	4.6	6.5
89.6	1.0	1.0	2.0	1.5	1.4	1.4	3.0	3.5
63.3	0.8	0.8	1.3	1.7	3.0	2.3	0.9	0.5
39.2	2.0	2.2	2.3	3.4	2.8	2.7	2.3	2.2
15.4	0.3	0.3	0.4	0.5	0.6	0.8	0.5	1.0
115.7	10.2	9.9	7.7	10.8	11.7	9.4	8.0	8.0
79.8	10.5	8.0	17.9	9.6	11.9	10.4	12.7	9.1
475.8	61.0	56.0	94.1	53.6	68.9	66.2	70.5	54.4
50.9	8.3	10.1	7.4	7.5	8.4	7.9	8.0	6.9
83.1	5.5	5.2	7.3	4.0	5.0	4.8	6.7	6.4
190.1	30.9	32.3	34.0	26.3	28.2	29.4	31.1	30.4
146.7	13.5	14.4	16.5	11.8	11.2	11.6	15.5	14.2
263.6	3.5	2.7	2.1	9.6	12.2	10.0	5.0	4.3
63.3	2.4	1.1	3.3	2.5	3.7	3.9	3.8	5.8
380.9	43.7	39.0	29.1	30.1	28.8	26.7	25.4	24.8
12.6	0.6	0.5	0.5	0.3	0.3	0.3	0.6	0.7
123.1	6.0	4.9	6.9	10.8	9.6	10.2	8.1	9.4
37.4	1.4	1.8	1.8	2.6	3.7	3.3	2.6	1.9
83.9	7.3	6.9	6.5	9.9	10.3	9.7	7.7	8.1
12.6	0.0	0.1	0.0	0.0	0.2	0.1	0.3	1.1
43.0	4.3	5.0	4.8	2.8	3.0	3.2	4.0	3.9
9.9	0.4	0.8	0.1	1.9	0.0	0.1	0.4	0.0
33.6	0.8	1.3	1.4	2.9	2.8	2.1	1.5	1.4
26.0	0.3	0.4	0.5	1.2	1.4	2.1	0.5	0.9
826.3	94.3	90.9	172.0	117.8	125.3	127.5	177.5	157.7
164.0	16.4	16.5	12.6	8.0	7.8	6.7	12.6	9.2
148.1	22.6	23.2	34.1	25.5	25.3	26.9	30.3	33.8
314.6	48.6	43.8	32.5	24.7	26.7	25.7	32.8	28.5
1201.7	100.3	88.6	51.6	25.6	29.2	32.9	15.4	14.8

27.3	0.4	0.8	0.3	1.4	1.9	2.1	0.4	0.7
72.5	0.2	0.2	0.1	13.2	0.4	0.1	2.5	1.8
99.1	16.1	18.3	13.0	10.2	10.5	10.8	12.1	11.2
3477.2	440.5	369.4	194.5	106.7	127.8	133.1	149.3	162.5
5579.4	95.7	91.6	151.8	340.1	500.8	543.5	270.4	429.5
25.3	1.8	1.5	2.7	2.1	2.8	2.9	3.9	3.3
39.3	3.0	3.4	1.0	0.3	0.1	0.0	0.1	0.0
30.4	4.5	5.5	8.9	4.6	5.7	3.3	4.5	4.4
274.8	29.3	31.4	32.4	36.3	44.8	44.0	40.0	48.6
132.7	5.5	5.7	7.8	15.6	17.0	14.4	10.6	8.6
102.0	10.2	10.4	14.9	13.8	18.1	16.9	19.3	18.5
178.9	35.6	31.8	30.7	20.6	18.8	18.5	24.1	19.3
1020.5	125.9	123.2	131.7	98.5	102.4	104.7	193.7	179.7
358.8	45.7	42.6	50.1	34.0	34.6	37.2	71.6	63.6
37.8	4.5	5.1	2.6	0.5	0.4	0.8	2.0	1.0
2093.1	491.1	379.6	366.3	402.0	485.0	470.2	218.4	321.3
11050.6	215.5	168.1	274.6	991.6	1021.8	1173.0	943.6	1365.8
957.7	248.5	219.9	189.6	138.1	131.5	125.9	151.2	181.7
228.0	53.3	51.5	37.2	27.3	27.8	28.9	33.6	24.1
821.6	84.1	76.4	114.4	135.8	162.5	178.8	145.9	174.6
42.2	4.2	3.4	7.4	6.1	6.3	6.1	11.1	10.7
80.0	2.0	2.1	2.9	6.7	12.6	14.7	0.8	2.9
1644.5	392.9	334.2	180.7	219.5	249.3	266.3	66.9	124.4
100.8	15.3	15.8	6.1	0.8	0.5	0.2	0.7	0.4
91.4	13.3	12.6	11.5	17.5	17.7	18.2	5.8	4.4
952.1	121.7	116.9	161.0	222.8	278.5	233.1	176.3	148.0
85.2	6.0	5.5	7.3	17.0	16.1	17.6	14.1	15.3
593.4	68.6	144.5	121.2	57.5	75.4	84.3	31.3	46.4
9.6	1.5	0.6	1.5	1.8	3.2	2.6	2.2	1.6
864.2	105.5	99.3	104.6	202.3	241.5	205.5	135.4	149.9
51.4	10.4	10.8	5.5	3.6	2.0	1.1	1.7	1.2
17.5	5.1	3.7	1.7	1.4	1.1	1.2	0.9	1.2

male-hind 3		
m_hi_MH4	**gene_name**	**gene_chr**
0.2	LOC100164798	NC_042496.1
4.9	LOC100165154	NC_042493.1
0.0	LOC103310029	NC_042494.1
0.0	LOC100166804	NC_042496.1
19.0	LOC100165462	NC_042495.1
0.0	LOC100169582	NC_042496.1
0.2	LOC100158949	NC_042493.1
0.0	LOC103309505	NC_042495.1
8.0	LOC100162815	NC_042496.1
0.5	LOC107883989	NC_042496.1
0.8	LOC100161127	NC_042495.1
33.7	Mvd	NC_042495.1
59.4	LOC100159664	NC_042495.1
1.2	LOC100163938	NC_042494.1
0.6	LOC107882164	NC_042495.1
1.3	LOC100162573	NC_042495.1
0.2	LOC103311408	NC_042494.1
0.7	LOC100572104	NC_042494.1
0.5	LOC100572448	NC_042495.1
31.4	Blvrb	NC_042494.1
2.4	LOC100161879	NC_042493.1
31.7	LOC100166744	NC_042495.1
3.8	LOC100165234	NC_042495.1
3.5	LOC100162856	NC_042495.1
5.4	LOC100163413	NC_042494.1
20.1	LOC100165274	NC_042495.1
49.4	LOC100165972	NC_042494.1
0.7	cp54	NC_042495.1
1.0	LOC100162683	NC_042494.1
0.0	LOC100166560	NC_042495.1
36.1	LOC100574505	NC_042493.1
2.7	LOC100162664	NC_042494.1
30.4	LOC100574136	NC_042494.1
14.3	Ipps	NC_042495.1
7.3	LOC100169498	NC_042494.1
6.6	LOC100160431	NC_042494.1
3.5	LOC100160076	NC_042495.1
1.2	LOC100169041	NC_042494.1
2.4	LOC100162264	NC_042493.1
0.6	LOC103307655	NC_042494.1
8.3	LOC100161003	NC_042495.1
9.9	LOC103311672	NW_021761939.1
52.6	LOC100161212	NC_042494.1
7.4	LOC100159865	NC_042496.1
6.9	LOC100159902	NC_042496.1
31.5	LOC100169605	NC_042494.1
15.6	LOC115034333	NC_042495.1
6.2	LOC100162731	NC_042494.1
4.1	LOC100575510	NC_042495.1
26.6	LOC100163305	NC_042494.1
0.9	LOC100569602	NC_042494.1
7.9	LOC100158803	NC_042493.1
2.5	LOC100163040	NC_042493.1
7.3	LOC100158932	NC_042494.1
0.1	LOC100159558	NC_042494.1
3.8	LOC100164200	NC_042495.1
0.0	LOC100571500	NC_042494.1
1.4	LOC100160352	NC_042495.1
0.5	LOC100569853	NC_042494.1
129.3	LOC100166089	NC_042496.1
8.3	LOC100165508	NC_042493.1
34.1	LOC100160499	NC_042494.1
31.7	LOC100161393	NC_042495.1
16.0	Elovl6	NC_042494.1

0.5	LOC100162877	NC_042495.1
1.6	LOC100574123	NC_042494.1
11.2	LOC100168638	NC_042494.1
161.8	LOC100163010	NC_042495.1
523.9	LOC100159357	NC_042494.1
3.3	LOC100162924	NC_042495.1
0.0	LOC100574710	NC_042494.1
4.6	LOC100168408	NC_042494.1
45.9	LOC100165720	NC_042495.1
13.3	LOC100167523	NC_042496.1
18.9	LOC100163144	NC_042494.1
18.3	LOC100167870	NC_042494.1
173.8	LOC100570401	NC_042494.1
61.2	LOC100161475	NW_021765209.1
0.6	LOC100571243	NC_042494.1
310.6	LOC100169524	NC_042494.1
1312.0	LOC100301633	NC_042494.1
160.2	LOC100160720	NC_042495.1
26.2	LOC100168697	NC_042495.1
165.9	LOC100160284	NC_042495.1
9.2	LOC100159885	NC_042493.1
4.1	ACYPI45536	NC_042494.1
129.8	Atpcl	NC_042495.1
0.3	LOC100162094	NC_042495.1
4.9	LOC100159025	NC_042494.1
173.9	LOC100160153	NC_042493.1
13.5	LOC100162951	NC_042495.1
51.4	LOC100167416	NC_042494.1
2.3	LOC100167746	NC_042495.1
174.7	LOC100160658	NC_042495.1
1.5	LOC100163328	NC_042494.1
1.8	LOC100571790	NC_042495.1

Table S2: Expression of mevalonate and nepetalactone pathway genes in hind legs and front legs of different sexual stages of *A. pisum*. RNA was extracted from aphid legs and sequenced, and the obtained reads were mapped onto the *A. pisum* genome version v3. Mean RPKM values are shown (n = 3). f-hl, hind legs of sexual females; f-fl, front legs of sexual females; af-hl, hind legs of asexual females; m-hl, hind legs of males.

Gene ID	Original gene annotation	f-hl	f-fl	af-hl	m-hl
100162815	acetyl-CoA acetyltransferase	1298.52	9.64	11.61	8.27
100165154	HMG-CoA synthase	5143.01	3.72	4.46	5.07
100165462	HMG-CoA reductase	1502.11	18.64	16.87	17.87
100163305	mevalonate kinase	374.42	37.29	28.55	25.64
100574505	phosphomevalonate kinase	1447.09	21.88	26.33	39.36
100163413	phosphomevalonate kinase-like	230.95	4.26	4.66	6.32
100158798	diphosphomevalonate decarboxylase	2093.95	29.54	36.26	31.61
100166744	isopentenyl diphosphate isomerase	2136.28	44.48	29.17	34.51
100144905	isoprenyl diphosphate synthase (ApIDS)	395.83	20.26	13.31	13.90
100158803	dolichyldiphosphatase 1 (ApPhos)	122.32	5.92	10.20	8.48
100162683	inositol polyphosphate 1-phosphatase	19.23	0.91	0.78	0.97
100165972	P450 (ApG8H)	3409.27	13.19	24.46	48.41
100160284	P450 reductase (ApG8H reductase)	804.93	91.65	159.00	162.16
100301633	farnesol dehydrogenase (ApHGO)	10416.49	219.39	1062.13	1207.17
100162094	retinol dehydrogenase	96.65	12.37	0.52	0.48
103310029	polyprenol reductase (ApISY)	140.59	0.00	0.03	0.05
100169582	GMC oxidase (ApNEPO)	28.53	0.00	0.00	0.03
100164798	GMC oxidase	3382.68	0.03	0.14	0.67
100168586	flavin reductase	266.86	31.77	33.21	32.24

Table S3: Signal peptide prediction with TargetP. Prediction probabilities signal peptides and the predicted cleavage sites are shown. mTP, mitochondrial transfer peptide; SP, signal peptide; CS, cleavage site.

**Protein**	**Prediction**	**SP**	**mTP**	**CS Position**
ApIDS	mTP	0.001316	**0.805817**	CS pos: 33-34
ApGES	-	0.000025	0.000236	
ApG8H	-	0.265424	0.010640	
ApRed	-	0.000022	0.000003	
ApHGO	-	0.002556	0.000248	
ApISY	-	0.113837	0.004443	
ApNEPO	-	0.167099	0.082896	
100164798	-	0.041727	0.000675	
100162683	mTP	0.001071	0.507994	CS pos: 23-24
100168586	SP	0.517806	0.004666	CS pos: 20-21
100162094	-	0.000450	0.002174	

Table S4: Sequences synthesized or amplified in this study.

Gene	Note	Sequence
ApIDS	N-terminal truncated<br><br>optimized for *E. coli*	ATGTCAACTGTTCGTGCCCCACCCGTCCCGCCCGTGATTACCGGTACCGCTGTCTCAAAGGACGAGACACG<br>TGACTTTATGGCGGTTTTCCCTGATGTAGTACGCGACCTCACCGATACCGGACGTAATCTGGACGTGCCGG<br>ACGTTACCAAATGGCTCGCCAAACTGTTACAATATAACGTGCCTGGCGGGAAGAAGAACCGCGGCCTGGCG<br>CTGGTCTTAAGCTATAAGATGTTAAGCTCGCCGGCAGATCAAACAGATGAGAATATCCGTCTGAGTTATAT<br>CTTGGGGTGGTGTGTGGAGATTCTGCAAGCGTATCAATTAGTTTTAGACGATATCATGGATAATGCAATCA<br>CGCGCCGTGGGCGTCCGTGCTGGTACCGCCATAATGATATTGGCCTGATGGCCGTTAATGATGGTGTCCTG<br>CTCGAACAGAGCATCTATCAACTCATCAAGAAGTACTTTAAGGACAAGCCTTATTATACTCATATCTTGGA<br>GCTTTTCTATGACGTTACCATGAAAACCTCTATGGGGCAATGTCTGGACATGCTTACCGCAAATTCTTTTA<br>AGAGTAAGAAGCTGGAGAAGTATACTATGGAGAATTACACAGCAATCGTCAAGTATAAGACGGCTTACTAT<br>TCGTTCTTCTTACCTGTGTGTTTAGCAATGCGCATGACTAATATTAATGACCCGGAGATCTTCCGTCAGGC<br>AAAGACCATCTTATTAGAGATGGGGCACTTCTTCCAAGTTCAAGATGACTTCCTGGACTGCTATGGCGATC<br>CGGACGTGATGGGTAAGATTGGAACCGACATTGAAGATGGCAAGTGTTCTTGGCTTGCGGTAGTCGCCCTT<br>CAGAAGGTTAATAGCGAGCAAAAGAAGATTATGGAAGACAATTATGGCATCGATAACCCCGCTAATGTAGC<br>AGTTATCAAGGACTTGTACGCGCAGTTGAAGTTACCCGACACTTTCCACTTGTACGAAGAGGAGAGCTATA<br>AGCTGATTTGTACTCACATCCAACAACTTAGCCGCGGCTTATCGCAGGATATGTTCTTCAAATTCCTGGAA<br>AAGATCTACAAACGCACCCTCTAA
ApGES	optimized for *S. cerevisiae*	ATGATGACTTACTCCGTTTCTTCTAATAAGAAGCATGACTTGTCTAACCGTAACATCTTTGGCAAGCTATT<br>AGCTCTTTTCTCATTAACTCCCTTTGTGATACTTTCTGGGTTCATCTCTTTGATATTATTTAGACGTGATT<br>TACACACCATCACGTTCTTCTTCGGTGTACTGTTAAATGAGATTTGTAACACGGTATTGAAGCACATTTTG<br>AGGGAACCCAGGCCTTTGGCAAGGAACACGAACCTATTATATAGCGAATACGGCATGCCAAGCTCTCATTC<br>CCAGTTTATGTGGTTCTTCGCCTCTTACATGCTATACTTTACGTTCATCAGGTTGCAGTACGCTAACAACA<br>AAGCATTCAAGGAGTTCTTTTGGAAGGTCGCTGGCGCAGTCAGCTGCATAGCTATAGCATGCATCGTTTCG<br>TACAGTCGTATTTTCTTGCAGTACCATACATGGAAGCAGGTTATATACGGTGCATTGTTTGGTATAATTAT<br>TGGAACCATTTGGTTCACAATTATCAATGTCGTATTGACTCCGTACTTCCCTACAGTTATCTCCTGGAAAA<br>TATCAGAGCTTTTCTTACTTAGGGACACTACTTTGATTCCTAACGTCCTTTGGTTCGAATATACAAATATC<br>AGACACGAGGCCGGAGCACGTGCCAGAAGAAGAAAGAGTATTTCCGCCAAGTCACAATAA
ApG8H	amplified from cDNA	ATGTTTGAATTCGTCTACGAACTGTTCGATCTGAAAATGCTTTTGGTCACCGCTTTTCTGGGTGCCATATA<br>CGTGTATTCCACATGGACCCACAGCCATTGGTCCAAGCTGGGCATATCCAGTCCGTCTGCCCCGGTGCCGT<br>TGTTCGGGCACGCGATGCCCTCCATGTTGGGACAGATGCACTTCATGGATGTGTTGCACAACCTTTACAAG<br>GAGCTGGGCGACCAAAGGTTTGGTGGCATTTACACAATGCGAACACCGCAGCTCCTCGTCAAAGACCCAGA<br>ACTAATAGGACACATACTGATCAAAGACTTCAACAATTTCACGGACCGCGGATTATACGCTGGCACACACA<br>CAAACCCGCTCAACAATAATATATTCTTCACACGAGGCGAACGATGGAAAACGATGCGGCAAAAGCTCAGT<br>CCCACATTCACGGCCAACAAACTGAAGTACATGAACGAACAAGTGAAGGAGTGCAGCGACGGTCTGCTGTC<br>GACTATCGGCAAGAACCTGGACGATGACGCCGGTCGGATCGAAATCCGCGAGATGATGGCCAAATACTCGA<br>CCGACGTGATCGGCAGCTGCGCGTTTGGCCTGAAGCTGGATGCCATCAACGATCCGGACTCGGAGTTCCGG<br>AAGCACGGAAAAACCGTTTTTCAGCCGTCGCTGAGGTCCAAGATCCGAGTGGCGGTCATATTCATGCAGCC<br>GTCCCTGCTGAGCATTTTCCGCGTGCATCACTACTCGCACCGCACCATCCGATTCTTCCACGACGCGTTCC<br>AGCAGACGATTGAATATCGGGAAAAGCACAACGAAGACCGCAAAGACTTTGTGCAGCATCTGATGAAGGCC<br>AGAGAAGATCTAGTGTTGAACCCGAATCTAAAACCCGAAGAAAAATTCACTGAAATGGATATTGTAGCGAA<br>CGCATATATTCTCTTCATCGCTGGTTTCGAAACAGTATCTACATCAATGAGCTTTTGTATGTATGAATTAG<br>CATTAAGAAAAGATGTCCAAGATAAAGTTCGAAAAGAAATATTGGAAGTTAAATCTAAGTACAATGGACAA<br>ATGAATAGTGAATGCCTTAACGAACTTCATTATATGGGCATGGTTATTAAAGAAACATTGAGAAAATATCC<br>TCCATTAGTGACATTAAATCGAGTTGTGACTAAGCCGTATGTAATACCAGGGACACAAATCAAGTTAAAAA<br>TAGGTACTAAAATTGTTGTTCCAGTACATGCCATTCACTACGATCCAAAATATTACTCTGATCCAGAGGCT<br>TTTGAACCAGATCGTTTTTCAGATGAAAACATACATAATATACAACCTAACACATATATGCCTTTCGGAGA<br>CGGTCCTAGATTTTGTATTGGCAAACGATTTGCTGAATTCGAAATGAAAATGGCCTTGTCCGAAGTGTTAA<br>CCAACTACGAAGTGATGGCATGTGATAAAACCCAAATTCCCATAAAATATGTTATCGGAAGTTTTGTGAAT<br>ATACCTGAAAGCGTTTGGTTAAAATTTAGGAAAGTGAATACTTAA
ApRed	amplified from cDNA	ATGGAGAATCCTGAAGGAGAGAAAATTGAATCAACTGTTGTTTCAGAAGAGCCATTGATTAGTGCTTTAGA<br>TATTGGTCTTTTGGTGGTTATTATAACAGTTGGATATTTTTGGTACATCAAAAGAGATAAAAAGTCAAGTT<br>CTTCAGAAAAAAAACCCTATACTATTCAGCCATCTTCGTTGAGTTCTATTGAGCAGACCTCCAATAGTTCT<br>TTTATAAAAAAACTTAAATCAACCGGTCGTAGTTTAGTAGTGTTCTATGGAAGTCAAACAGGGACTGCAGA<br>AGAATTTGCTGGAAGAATAGCCAAAGAAGGGGCCAGATATAAAATGAAAGGAATGGTAGCCGATCCTGAAG<br>AATGTGATATGGCAGACTTGGTAGAAATGAAAGAAATACAAAAGTCATTGGCAATTTTTTGTATAGCAACA<br>TATGGTGAGGGAGACCCGACTGATAATGCTATGGATTTCTACGAATGGCTTCAAAATGGTGACGCCGACCT<br>AGAAGGATTGAATTATGCAGTATTTGGATTGGGAAATAAGACTTACGAGCATTATAACGAAATTGCTATTT<br>ACATTGATCAACGTTTGGAAGAATTGGGTGCTACTAGAGTTCATGAAATTGGTCTAGGAGATGATGATGCC<br>AATATTGAAGATGATTTTGTTTCTTGGAAAGAAAAATTATGGGATAGTGTTTGCTCCCATTATGGTATTGA<br>AGAAACTGGCGAAGAAAGTAATATTAGACAATACAAATTAGTAGACTGTTCCGAAGTTTTACCAGAGCGAA<br>TATTTTCGGGTGAAATATCACGACTTAAATCTTATGAAAACCAAAGATTCCCATTTGATGTTAAGAACCCA<br>TATTTATCTAAAATATCAGTTAATCGTGAGCTCCACAAGTCAGGTGATCGCTCTTGTATGCATATTGAGTT<br>TGATATTGATGGATCAAAGATGAGGTATGACACAGGAGATCATGTGGCTGTCTATCCCAAAAATTCTTCTG<br>AATTAGTAGAGAAAATTGGAGAATTGTTAAATGCAGATCTAGACACTGTATTTTCATTGTTGAACACTGAT<br>GAGGAATCCAGTAAAAAACATCCATTCCCTTGTCCTTGTACTTATCGAACAGCTTTAACTTATTATTTGGA<br>TATAACTTCAAATCCACGCACACATATTATGAAAGAATTAATAGAATATGCAAGTGATCCAAAGGATCAAG

		AAAAATTAAAGCTTATGGCAAGCTCAACCCCAGAGGGCAAAAAAGAATTTCACGAATGGATATTGCGTGAC<br>AACCGTAATATTGTTCATATTTTGGAAGATTTACCGAGTGTCAAACCAGATTTGGACCATTTATGTGAACT<br>ACTCCCACGATTGCAGTGTCGTTATTATTCAATATCCTCGTCACCAAAAGTGTACCCAAAATCTATTCATA<br>TTACAGCAGTTCTAGTAGAATACACTACTCCTACTAATCGAGTCAATAAAGGTGTTGCAACTAACTTATTA<br>GCCCAACTGAAACCAACCAATGACGAACTTCTACAACCTACTATACCTATTTATATTAGACGATCTCAATT<br>CAGATTGCCCCCTAAGAGTCAAACTCCAATTATAATGATTGGTCCCGGTACAGGATTAGCACCATTCCGAG<br>GTTTTATACAAGAAAGAGATTATGCACGTAAAGAAGGTAGAGAAATAGGAGAAATGGTTCTGTACTTTGGA<br>TGTCGAAAAAAGGATGAAGACTTCATTTATGAAAATGAGTTACAAGAATATGTTGCTAATGGAACCTTAAC<br>AAAGCTACATTTGGCATTTTCGCGTGATCAACCTGAAAAGCAGTATGTGACACATTTGTTAGAACAAAATG<br>CTGATGAACTGTGGAATATTATTGGTGAAAAAAATGGTCATTTATATGTTTGTGGTGATGCAAGGAGCATG<br>GCAAAGGATGTGCATAGTATTATTGAAAAAGTTGTCATGGAAAAGGGTCAAATGACCAATAGTCAGGCACT<br>CAATTATGTTAAAAAAATGGAACAACAGAAAAGATATTCTGCTGATGTGTGGAGCTGA
ApHGO	optimized for *E. coli*	ATGGAGAAATGGAATGGCAAAGTCGCTGTGGTCACAGGCGCCTCGAGTGGAATTGGCGAGGAGACCTGTCG<br>CCAGCTTGTGGAGCGCGGAATGATCGTTGTAGGGTTCGCACGCCGCGAGGACAAACTCCAGGAGCTGGAGA<br>AGGACTTAAAAGGGAAGCTGGGAAAGTTTTATTACGTGAAAGTAGATCTTTGCTCCGAGGAAAATATTATG<br>GAAGCATTCAACTGGGTAAAGAGCACTCTGAAATCGGTGGATGTTCTGGTCAATAATGCCGGCGTTCTGAA<br>GAAGAGTGATTTACTGGGCAACACTAACGATTGGAAACAGATGTTTGATACGAACGTTATGGGTCTGAATA<br>TTTGCTCACGTGAAGCCATTAAGATCATGGAAGAAATCCAAATTAAAGAGGGCCACATTATTAACATCAAT<br>AGTGTTGGTGGACACTACCAGTTCCAATTTGTAAAGGATTTCTCAGTCTACTGCGCCACGAAACACACCGT<br>GACCATCATTACAGAAAGCCTGCGCGAACTGATGGGTATGAAGAATCTTCCGGTCCGTGTTACGTCCATTA<br>GCCCGGGAGCGGTGGACACTGAGATGACTCTCGAGTTCTCAAAGATGGAAGGGTTCAAGATGCTTAAGAGC<br>ATCGACATTGCCGAGGCGATTTTATATGCCCTCAGCGCGCCGCAACGTGTAAACGTAGCGGAAATTATTAT<br>TCGTCCCACCGGCGAGAACACTGCGGGCTTGATTAAGAATTTCGTATGA
ApISY	amplified from cDNA	ATGATGGACGTCGTAAACGTTATGTTCGTCGGTATGACGTTCGTGTTCGTGGTTGTGCCGTTGGTGCTCAA<br>GTCGATGGACGGGACTCGATTGCCGGTCGCTGTGGCCCGATTGTACGCGTACGGCAAGATCACGGGCGGCG<br>CGAAGCCGTCCGGCGTGCTGAGCGTGCCCAAGCGGTGGTACAAGCACTTTTACGCATTCTCATTGGCCCTG<br>TCACTGATGGCAGCGCTGGCGCTGTCAGACGAGTATGCGGCGGGAGCCCCTTGGCCGTCGGCCGCGGCGTG<br>GTGCCGGCGGTGGCTGTGGGACCCGGTCCGCCGACCGAAGTCGTCGTACAGCTCGGCCGCCGCGTCCACGG<br>CCGCCGGCATGTTCGTGCTACAGTGCGCCAGGCGGACGTACGAGACGTTCCACGTGAACGTGTTCTCGGAC<br>ACGGCGGTTGGGCTGTGGTATTACGCGTCCGGTTACATGCACTACATAGGCGCCATCGTCACCGTGCTGGC<br>CGAGGCGCCGGTCGCAGTGGCGGCCGGGGAGCCACAGTCCAGCGGCGCCTGGGAGCCTGTCCGGTTGGCCG<br>GGGCCCTGCTGGTGTTTGCCTGGGCATATCGCGAACAGTGGCGGGCCAACGTGGCACTGGCCGAGGCCCGG<br>AAGCGCGGAGGTCAGGTGGTCACGCACGAGCACATTATGCTCACCGGCGGACTTTTCGACCTCGTGTCTAG<br>TCCACAGATGCTCACCGAGGTGGTCTTGTACGGCGCTTGGTACGCCGTGCTGTGGGGCACCACCGGATGGA<br>AGTACGTGATCGCGTTCGTCTGGGGAAATCAGTTCGAGATAGCACTCATCAGTCACCGGTGGTACCAGGAC<br>AAGTTCCCGAACTACCCACGAGAAAGGAAAGCTATCATACCGTATATTCTGTAA
ApNEPO	optimized for *E. coli*	ATGGCATCTCTTATCAGTGGGGCAATGAGCAGCGCAGCGTGGACCGGCGCGGGTATGATTCCTGTGTTCGT<br>GGTAGGACTTGCATACCTGCGTTATTCGATGTATGACCCGGAGTCCCGTGTGGTAGATGTGCTGGAAGTGC<br>GCGACGAGTACGACTTCATTGTGGTCGGCGCCGGTAGCGCAGGTGCAGTCATCGCAAATCGCTTGTCCGAA<br>ATGCAAAATTGGACCGTGTTAGTCTTAGAGGCCGGTGGTGACGAGACCGAAATTAGTGATGTTCCTTCTTT<br>CGTAGGCTATTTACAGCTTTCGGACATGGACTGGCAATATAAAACAGCCCCGCCGAGTAGTGATAATCCAT<br>ATTGCCTGGCGATGGTTCACGACCGTTGTAACTGGCCTCGTGGTAAGGTGCTGGGTGGCTCGTCGGTCTTG<br>AATGCAATGGTGTATGTCCGCGGTAATCAACGTGACTATGATATGTGGGCGGCCGCGGGAAATCCCGGTTG<br>GGCGTACGCTGACGTCTTGCCATATTTCCTCAAATCGGAAGATAATCGCAATCCGTACTTGGCGCGTACGA<br>AATATCATGCCCGTGGTGGCTACCTGACGGTCAGCGAGGCCCCGTGGCGCACGCCTCTCGCCACAGCTTTT<br>GTTGCGGCTGGTGAGGAGCTTGGTTACCAAAATCGCGATATCAACGGCCAATACCAAAATGGATTCATGCT<br>CACCCAAACGACGACCCGTCGTGGTTCCCGCTGTTCAACTGCCAAGGCGTTTCTGCGTCCTATTCGTCTGC<br>GTCCGAATATTCACGTGTCGATGCACAGTCAAGTAACTCGTATTCACTTCAGCGGTGGTAACGGCGGCAGC<br>GACAAACTTCGTGCAACAGGTGTCACTTACCTTCGCAATGGGAAACGCCGTACTGTTACTGCTCGCAAAGA<br>GGTAATCTTGAGTGCAGGGGCGATTGGCAGTCCCCAATTGCTGATGGTCAGCGGAGTAGGACCCGCTGATC<br>ACCTTACTGAACTGGGCATTAAGCCAGTCGTAGATCTGAAGGTAGGGCATAACTTACAGGATCACGTAGGC<br>TTAGGCGGTCTCACTTTCCTTATTGATGACCCAATCACGTTCAAGAAGTCACGCTTCACCTCAGCCTCGGT<br>GGCCCTGGATTACATCATGAACGAGCGTGGTCCTTTGACCTCGAGTGGAGTCGAGGGTCTCGCGTTCGTAA<br>ACACCAAGTACGCAGACCCGTCCGGCGAGTTTCCGGATATTCAGTTCCACTTTGCACCCTCGTCAGTCAAT<br>AGCGATGGGGACCAAATTCGCAAAATCACCGGTCTGCGCGACGCGGTTTACAATACAGTATACAAGCCATT<br>GGTGAATGCCGAAACATGGACTCTCCTTCCTCTCTTATTGCGTCCAAAGAGCTCGGGTTGGGTGCGCCTTA<br>AGAGTAAGAACCCACTTGCCCACCCCATCATCGAGCCCAATTACTTTGCCCACCGCGAGGACGTCCAAGTT<br>CTCGTCGACGGGATTCGCATCGCATTCAACGTAAGCAATACCGCCGCGTTTCGTAAGTACAATTCCCGTCC<br>ACTTTTAACCCCGATGCCAGGCTGTAAGAAGTTTGAGCTGTTCAGTGATGAGTACTGGGAATGCGCTCTGC<br>GCCACTTCACGTTTACCATCTACCACCCTGCGGGAACGTGTAAGATGGGCCCGGACACGGACCCCGACGCA<br>GTGGTTGACCACCGTTTACGCGTGCGTGGGATCGATGGCCTGCGTGTGATTGACGCCTCTATTATGCCTAA<br>TATCATCAGTGGTAATCCTAATGCCCCAGTGATCATGATTGGCGAGAAGGGTGCCGACATGATTAAGAAGG<br>ACTGGTCAAGCTAA
100164798	optimized for *E. coli*	ATGTCGGGAGTTGAAGTCATCCCAATTGGAGCAATCGCCGGAGCCGCCAGCCAGGTGGCCTGGTTTCTTCC<br>AGTTCTGGTTGCCGCCATCGCGTATTTCCATTATGAGGTCAACGATCCGGAGAGTCGCATTATTGATCAAC<br>CGGGCAATCTCATTTTAGACCAATACGATTTTATCATTGTGGGTGCAGGCAGCGCCGGTGCGGTCTTAGCA<br>AACCGTCTTACCGAGGTTGAGGACTGGTCGGTGCTTCTCATCGAAGCTGGCGGTGATGAGACGGAGATTTC<br>CGACGTGCCCCTTCTTGCCGCGTATTTGCAGTTGACTCAATTGGACTGGCAATATAAGGCGGAGCCCCAAG<br>ACACCGCCTGCTTAGCAATGAAAGATCAGCGCTGCAATTGGCCACGCGGTAAGGTTTTGGGCGGCAGTTCT<br>GTTTTGAACTATATGATGTACGTACGCGGTAATCAGATGGATTACGATAGTTGGTTAAAGCAAGGCAATCC<br>GGGCTGGGGTTACAACGACGTCCTTTATTATTTTAAGAAGTCTGAGGATAATCGTAACCCATATCTTGCTC

		GCACGCCCTACCACAGTATGGGCGGTTATTTAACAGTGTCCGAAGCACCGTATAAGACCCCTCTGGCAGAC<br>GCGTTTGTTCGTGCGGGCCAGGAGATGGGATACGACATCCGTGATATCAATGGCGAACGCCAGACAGGCTT<br>CATGATCCCTCAAGGCACCATCCGTCGCGGCGCCCGTTGCAGCACGGCAAAGGCCTTCTTACGCCCCGCCC<br>GTTTGCGTAAGAATTTACACGTCTCTATCAATGCCCACGTGACTCGCGTTGCGATTAACCCTGAGACCAAG<br>GTGGCATTTGGCGTGGAGATGATCAAGGACAACACGCGTCATTTCATTCGTGCAAATAAAGAGGTGTTACT<br>GTCCGCCGGCTCCATCTCATCGGCCCAACTTCTTATGCTGTCAGGCATTGGGCCGAAGAACCACCTTACTG<br>AAATGGGCATTCCGGTCTTGGCCGACTTAGATGTAGGAAAGAACCTGCAAGACCACGTAGGGTTAGGTGGC<br>TTGGCCTTTCTGATCAATAAGGAAGTTAGCTTGACCCAGGAACGCGTGGAAAATGTACAAACCGTGTTGAA<br>TTACGCCACCATGGGTGACGGACCGTTGACCGTCATGGGTGGCGTAGAGGGGTTAGCCTTTATCAATACAA<br>AATACGCGAACCAGAGCGTGGACACCCCTGATATTGAGCTCCACTTTGTTTCCGGAAGTACCAATAGTGAT<br>GGCGGTGTCCAATTATGGAAAGCCCACGGCTTAAAAGAGGAATTTTATAAGGCGGTGTACGAGCCAATCAA<br>TAACAAGGACGTGTGGTCTGCTATCCCCATGCTGTTGCGCCCGAAGAGCCGTGGAGAGATTCTTCTTCGCA<br>GCACCGACCCGTCCATGTACCCACGTATTCTGCCTAATTACCTCACCGTGCAAGAGGACGTCGATACCTTA<br>GTTGAGGGTGTGAAGTTTGTGGTGGCAATGTCTCGTACGACCCCATTCCGCCGTTATGGTTCCCGCCTGCA<br>CGATATTCCGTTCCCTGGTTGTGCGGCGGTGCCCCGTTTCACGGACGCGTATTGGGAGTGTATGGTCCGTC<br>ATTACACTGTGACCATCTATCATCCAGTGGGTACGGCAAAGATGGGCCCTGAGTGGGACAAGACAGCCGTA<br>GTGGACCCGCGCCTGCGCGTCTACGGTATTCACGGCCTTCGTGTAGTGGATGCCAGCATCATGCCCACCCT<br>TGTAAGCGCCAATACCAATGCACCTGTCATCATGATCGCTGAGAAGGCTGCTGACATGATCAAGGAGAAGT<br>GGCTGGGACGTAAGCGTTGA
100168586	optimized for *E. coli*	ATGAAGAAGATTGCGATTTTCGGGGCAACCGGTATGACAGGACTGTGTACCGTTGAGGCCGCATTAAAGCA<br>AGGGCTCGAGGTGCGCGCCTTGCTTCGTGACCCCAGCCGCATGCCCGAAGAGCTGCGCAAGCAGGTTGAGG<br>TAATCACAGGTGACGTCTTAGTGAAAGAGGATGTTGACAAGGTGGTTGAGGGCCGCGACGCGATCGTCGTC<br>ACACTGGGGACACGTAACGATTTAGCACCAACTACGATCATGTCAGAGGGTCTTCGTAATATTCTGTCGTC<br>AATGGAAAAGAACAATGTTAAGATCGTTAGCGTATGTCTTTCTACGTTCTTATTCTACGATAAACCCAAGG<br>TGCCCGCGATGTTCCATGGCATTAACGACGACCACGAGCGCATGTTACATCTCCTTCAGGCGGCAGAGAGC<br>CTGGACTGGATTGCTGTAATGCCGCCGCACATCGCGGGTACGCCGAGCGGGGACTACTCAGTTGAGATTGG<br>CTCAAGTCCGGGGCGTGCGATTAGCAAGTATGACTTGGGAAAGTTTATGATTGAGTGTCTTAGCAAACCAG<br>ATTATTATAAGCAACGCTGTGGTCTGGCTACGAAAGTTCCAGCGCCGTGA
100162094	optimized for *E. coli*	ATGGAGTACTTCCTGCCGAACCGCTGTACGTCAACTGTTCGTTTAGACGGCAAAACAGTCGTAGTTACTGG<br>CTGCAATACGGGCATTGGGAAGGAGACGGCAACGGAGTTCTACAAGCGCGGCGCACGCGTGATTATGGCTT<br>GTCGTTCGGCTAGCCGCACGCAAGATGCTATTGAATCGATCAAGAATCAAACGGAAGGCGATAACAACGTG<br>GGTGAGTTGGTCTTTAAGCACTTGGAGCTTAGCTTTCTGGCATCCGTACGTAAGTGCGCGAAGGAGATCCT<br>GCACACGGAGAAACGCATCGACATTTTAGTCAACAACGCCGGCATTATGATGTGCCCCAAGACGCTTAGTG<br>AGAATGGTATCGAGCTGCATTTGGCCACTAATCATCTGGGCCACTTCCTTTTCACTCTGTTGTTATTACCG<br>CGCATCCTGAAGAGCGCGCCCGCACGCATTATCAACGTCACATCCCTGGCTCACAAATGGGGAGATCAGAA<br>GATGCACTTTGATGACATTAATCTTGATAAGGACTACACCCCGTCAGGCGCGTACGGACGCTCAAAGCTCG<br>CCAATATCCTCTTTACGGTGGAGCTTGCTAAACGCCTGAACGGGACGGGCGTAACGGTTTACGCTGTAAAT<br>CCCGGTATTGTACATACCGAGCTCTCGCGTTATGTCGACCAAACTATTTTCCCAGGTGCGTCCTGGCTTTA<br>TAACTCATTCACGAAGATTGCAGTGAAGACTCCTCAACAAGGCGCACAAACGACCTTGCACTGTGCGCTTG<br>ATGAGAAATGTGCTGGCGAAAGCGGCTTGTACTACAGTGACTGTAAGGTTCTTGAGCCTGAGCCAGTAGCA<br>AAAGACGAAGAAGTCAGCGCCCAATTGTGGGATACTTCCTGCGCTTTTGTTAACCTGGAACCGTCTATTGA<br>TCCTTTTAAGCCCGAATCGGACGATGTCTCCATTTGA
100162683	optimized for *E. coli*	ATGTTAAGCCGTCGCTGCGCATATCGCTCGCAGGTTTCTCGTGGCGCGCACGGCGTTTGCACGATGTTTAG<br>CTCGGATGGCATGACATGTCCTCCTGATGCTGATGGTACGGCAGCCTGTGGCCATCGGCGTCGCTGCTCAG<br>GTCTGATCGGTTGCGCATTGCTCGCCTCTGAGGCCGCTGCGCACGTCGCTCGTCTTTGTCGCTCAAATCCG<br>CGCCTGCTGTCCATGCTGGTGCAGGAGAAATGCGGTGATGATGCCAATAGTTCACGCTTTGCTCATGATTT<br>CAAAACCCTGGCTGATGTTCTGGTTCAACGCGTGGTATCTAAACGTATTGGCCGCCAATTTCCGGAGTTAG<br>AAGGTAACGTGTATGGGGAGGAAAATGATACCATTCAGAATAATAAAGGCCAAGACGTCACTATTCAGGTT<br>GGCGAAACGGTTGAGGAAACGCTGCAGTGCCTTACTAAAGCGCTCGGCGATGACTTGGACAGTGCCAAGAG<br>CTTGGCCGAAGCGGCCCATAAAGAGTTTGAAAGTAAGGATCTCAACGTGGATTGCTATCCGCCAGAAGAAG<br>AGAACTTAGATCTGGAAAAAATCGGGATTTGGATCGACCCGATTGATTCGACCAACGAGTATATCAATGGA<br>AATGTGGACACCATCAATGAATATGGCTTTCATTCGTCAGGCCTGCATTGCGTGACTGTGAACATTGGTCT<br>GTTCGATAAATATTCTGGTAAACCCATTGCGGGGGTGATTAATCAGCCTTTCTTCGAATTCGACGCGGTCA<br>ACGGCTGGAACGGCCGCTGTTACTGGGCATACTGCGACGGCCAGAAATCGCTGAACAGTCTCCCAGAATTT<br>ATTAGCTGTAATCAGGAATTAGTGGTCACTTCCAACAGCGAAACTGATGCGGCTAAAGCCGCATTACGTCG<br>TTCCGGTTATACCGTTGCAACCGCGAGTGGAGCGGGCTATAAAATGCTTTGTGTGGCGCTGGGAATCGTGA<br>AATGTTATGCGCTTACCAAAGACTCAACGTACGCCTGGGACACCTGCGCGGCTCATGCTATGTTGGCGTCG<br>CAGGGCGGAAAAGCCTGTCAGTGTAACACAGCAGATGGGCCTTTAACTTATCGTCCGAAAACTGCGGGTGG<br>CGGTCGGGCCCATTGCAATGCAGCCGGCGTGATTGCTTCCCGTGATCCACAAACGGTTGACCGTGTCCATG<br>CCCTGTTATCCCCGTTACATCTGCATTGCAGCAGTAGTTCATAA

Table S5: Prediction of transmembrane domains by DeepTMHMM. S, signal peptide; I, inside cell/cytosol; M, alpha membrane; B, beta membrane; P, periplasm; O, outside cell/lumen of ER/Golgi/lysosomes.

Protein	Transmembrane domain prediction
ApIDS	MNKMLTFTRALSRRSAFLLSDSAAVRENCFRSMSTVRAPPVPPVITGTAVSKDETRDFMAVFPDVVRDLTDTGRNLDVPD<br>VTKWLAKLLQYNVPGGKKNRGLALVLSYKMLSSPADQTDENIRLSYILGWCVEILQAYQLVLDDIMDNAITRRGRPCWYR<br>HNDIGLMAVNDGVLLEQSIYQLIKKYFKDKPYYTHILELFYDVTMKTSMGQCLDMLTANSFKSKKLEKYTMENYTAIVKY<br>KTAYYSFFLPVCLAMRMTNINDPEIFRQAKTILLEMGHFFQVQDDFLDCYGDPDVMGKIGTDIEDGKCSWLAVVALQKVN<br>SEQKKIMEDNYGIDNPANVAVIKDLYAQLKLPDTFHLYEEESYKLICTHIQQLSRGLSQDMFFKFLEKIYKRTL<br>IIIIIIIIIIIIIIIIIIIIIIIIIIIIIIIIIIIIIIIIIIIIIIIIIIIIIIIIIIIIIIIIIIIIIIIIIIIIIIII<br>IIIIIIIIIIIIIIIIIIIIIIIIIIIIIIIIIIIIIIIIIIIIIIIIIIIIIIIIIIIIIIIIIIIIIIIIIIIIIIII<br>IIIIIIIIIIIIIIIIIIIIIIIIIIIIIIIIIIIIIIIIIIIIIIIIIIIIIIIIIIIIIIIIIIIIIIIIIIIIIIII<br>IIIIIIIIIIIIIIIIIIIIIIIIIIIIIIIIIIIIIIIIIIIIIIIIIIIIIIIIIIIIIIIIIIIIIIIIIIIIIIII<br>IIIIIIIIIIIIIIIIIIIIIIIIIIIIIIIIIIIIIIIIIIIIIIIIIIIIIIIIIIIIIIIIIIIIIIIIII<br># ApIDS Number of predicted TMRs: 0
ApGES	MMTYSVSSNKKHDLSNRNIFGKLLALFSLTPFVILSGFISLILFRRDLHTITFFFGVLLNEICNTVLKHILREPRPLARN<br>TNLLYSEYGMPSSHSQFMWFFASYMLYFTFIRLQYANNKAFKEFFWKVAGAVSCIAIACIVSYSRIFLQYHTWKQVIYGA<br>LFGIIIGTIWFTIINVVLTPYFPTVISWKISELFLLRDTTLIPNVLWFEYTNIRHEAGARARRRKSISAKSQ<br>OOOOOOOOOOOOOOOOOOOOOOOMMMMMMMMMMMMMMMMMMMMMIIIIMMMMMMMMMMMMMMMMMMMOOOOOOOOOOOOO<br>OOOOOOOOOOOOOOMMMMMMMMMMMMMMMMMIIIIIIIIIIIIMMMMMMMMMMMMMMMMMMMMMMMMOOOOOOOOOMMMM<br>MMMMMMMMMMMMMMMIIIIIIIIIIIIIIIIIIIIIIIIIIIIIIIIIIIIIIIIIIIIIIIIIIIIIIIIII<br># ApGES Number of predicted TMRs: 5
ApG8H	MFEFVYELFDLKMLLVTAFLGAIYVYSTWTHSHWSKLGISSPSAPVPLFGHAMPSMLGQMHFMDVLHNLYKELGDQRFGG<br>IYTMRTPQLLVKDPELIGHILIKDFNNFTDRGLYAGTHTNPLNNNIFFTRGERWKTMRQKLSPTFTANKLKYMNEQVKEC<br>SDGLLSTIGKNLDDDAGRIEIREMMAKYSTDVIGSCAFGLKLDAINDPDSEFRKHGKTVFQPSLRSKIRVAVIFMQPSLL<br>SIFRVHHYSHRTIRFFHDAFQQTIEYREKHNEDRKDFVQHLMKAREDLVLNPNLKPEEKFTEMDIVANAYILFIAGFETV<br>STSMSFCMYELALRKDVQDKVRKEILEVKSKYNGQMNSECLNELHYMGMVIKETLRKYPPLVTLNRVVTKPYVIPGTQIK<br>LKIGTKIVVPVHAIHYDPKYYSDPEAFEPDRFSDENIHNIQPNTYMPFGDGPRFCIGKRFAEFEMKMALSEVLTNYEVMA<br>CDKTQIPIKYVIGSFVNIPESVWLKFRKVNT<br>IIIIIIIIIIIIIIIIIIIIIIIIIIIIIIIIIIIIIIIIIIIIIIIIIIIIIIIIIIIIIIIIIIIIIIIIIIIIIIII<br>IIIIIIIIIIIIIIIIIIIIIIIIIIIIIIIIIIIIIIIIIIIIIIIIIIIIIIIIIIIIIIIIIIIIIIIIIIIIIIII<br>IIIIIIIIIIIIIIIIIIIIIIIIIIIIIIIIIIIIIIIIIIIIIIIIIIIIIIIIIIIIIIIIIIIIIIIIIIIIIIII<br>IIIIIIIIIIIIIIIIIIIIIIIIIIIIIIIIIIIIIIIIIIIIIIIIIIIIIIIIIIIIIIIIIIIIIIIIIIIIIIII<br>IIIIIIIIIIIIIIIIIIIIIIIIIIIIIIIIIIIIIIIIIIIIIIIIIIIIIIIIIIIIIIIIIIIIIIIIIIIIIIII<br>IIIIIIIIIIIIIIIIIIIIIIIIIIIIIIIIIIIIIIIIIIIIIIIIIIIIIIIIIIIIIIIIIIIIIIIIIIIIIIII<br>IIIIIIIIIIIIIIIIIIIIIIIIIIIIIII<br># ApG8H Number of predicted TMRs: 0
ApRed	MENPEGEKIESTVVSEEPLISALDIGLLVVIITVGYFWYIKRDKKSSSSEKKPYTIQPSSLSSIEQTSNSSFIKKLKSTG<br>RSLVVFYGSQTGTAEEFAGRIAKEGARYKMKGMVADPEECDMADLVEMKEIQKSLAIFCIATYGEGDPTDNAMDFYEWLQ<br>NGDADLEGLNYAVFGLGNKTYEHYNEIAIYIDQRLEELGATRVHEIGLGDDDANIEDDFVSWKEKLWDSVCSHYGIEETG<br>EESNIRQYKLVDCSEVLPERIFSGEISRLKSYENQRFPFDVKNPYLSKISVNRELHKSGDRSCMHIEFDIDGSKMRYDTG<br>DHVAVYPKNSSELVEKIGELLNADLDTVFSLLNTDEESSKKHPFPCPCTYRTALTYYLDITSNPRTHIMKELIEYASDPK<br>DQEKLKLMASSTPEGKKEFHEWILRDNRNIVHILEDLPSVKPDLDHLCELLPRLQCRYYSISSSPKVYPKSIHITAVLVE<br>YTTPTNRVNKGVATNLLAQLKPTNDELLQPTIPIYIRRSQFRLPPKSQTPIIMIGPGTGLAPFRGFIQERDYARKEGREI<br>GEMVLYFGCRKKDEDFIYENELQEYVANGTLTKLHLAFSRDQPEKQYVTHLLEQNADELWNIIGEKNGHLYVCGDARSMA<br>KDVHSIIEKVVMEKGQMTNSQALNYVKKMEQQKRYSADVWS<br>OOOOOOOOOOOOOOOOOOOOOMMMMMMMMMMMMMMMMMIIIIIIIIIIIIIIIIIIIIIIIIIIIIIIIIIIIIIIIIII<br>IIIIIIIIIIIIIIIIIIIIIIIIIIIIIIIIIIIIIIIIIIIIIIIIIIIIIIIIIIIIIIIIIIIIIIIIIIIIIIII<br>IIIIIIIIIIIIIIIIIIIIIIIIIIIIIIIIIIIIIIIIIIIIIIIIIIIIIIIIIIIIIIIIIIIIIIIIIIIIIIII<br>IIIIIIIIIIIIIIIIIIIIIIIIIIIIIIIIIIIIIIIIIIIIIIIIIIIIIIIIIIIIIIIIIIIIIIIIIIIIIIII<br>IIIIIIIIIIIIIIIIIIIIIIIIIIIIIIIIIIIIIIIIIIIIIIIIIIIIIIIIIIIIIIIIIIIIIIIIIIIIIIII<br>IIIIIIIIIIIIIIIIIIIIIIIIIIIIIIIIIIIIIIIIIIIIIIIIIIIIIIIIIIIIIIIIIIIIIIIIIIIIIIII<br>IIIIIIIIIIIIIIIIIIIIIIIIIIIIIIIIIIIIIIIIIIIIIIIIIIIIIIIIIIIIIIIIIIIIIIIIIIIIIIII<br>IIIIIIIIIIIIIIIIIIIIIIIIIIIIIIIIIIIIIIIIIIIIIIIIIIIIIIIIIIIIIIIIIIIIIIIIIIIIIIII<br>IIIIIIIIIIIIIIIIIIIIIIIIIIIIIIIIIIIIIIIII<br># ApRed Number of predicted TMRs: 1
ApHGO	MEKWNGKVAVVTGASSGIGEETCRQLVERGMIVVGFARREDKLQELEKDLKGKLGKFYYVKVDLCSEENIMEAFNWVKST<br>LKSVDVLVNNAGVLKKSDLLGNTNDWKQMFDTNVMGLNICSREAIKIMEEIQIKEGHIININSVGGHYQFQFVKDFSVYC<br>ATKHTVTIITESLRELMGMKNLPVRVTSISPGAVDTEMTLEFSKMEGFKMLKSIDIAEAILYALSAPQRVNVAEIIIRPT<br>GENTAGLIKNFV<br>IIIIIIIIIIIIIIIIIIIIIIIIIIIIIIIIIIIIIIIIIIIIIIIIIIIIIIIIIIIIIIIIIIIIIIIIIIIIIIII<br>IIIIIIIIIIIIIIIIIIIIIIIIIIIIIIIIIIIIIIIIIIIIIIIIIIIIIIIIIIIIIIIIIIIIIIIIIIIIIIII<br>IIIIIIIIIIIIIIIIIIIIIIIIIIIIIIIIIIIIIIIIIIIIIIIIIIIIIIIIIIIIIIIIIIIIIIIIIIIIIIII<br>IIIIIIIIIIII<br># ApHGO Number of predicted TMRs: 0
ApISY	MMDVVNVMFVGMTFVFVVVPLVLKSMDGTRLPVAVARLYAYGKITGGAKPSGVLSVPKRWYKHFYAFSLALSLMAALALS<br>DEYAAGAPWPSAAAWCRRWLWDPVRRPKSSYSSAAASTAAGMFVLQCARRTYETCHVNVFSDTAVGLWYYASGYMHYIGA<br>IVTVLAEAPVAVAAGEPQSSGAWEPVRLAGALLVFAWAYREQWRANVALAEARKRGGQVVTHEHIMLTGGLFDLVSSPQM<br>LTEVVLYGAWYAVLWGTTGWKYVIAFVWGNQFEIALISHQWYQDKFPNYPRERKAIIPYIL

	OOOOMMMMMMMMMMMMMMMMMMMIIIIIIIIIIIIIIIIIIIIIIIIIIIIIIIIIIIMMMMMMMMMMMMMMMMMMMMMM<br>OOOOOOOOOOOOOOOOOOOOOOOOOOOOOOOOOMMMMMMMMMMMMMMMMMMMIIIIIIIIIIIIIMMMMMMMMMMMMMMM<br>MMMMMMOOOOOOOOOOOOOOOOOOOOOMMMMMMMMMMMMIIIIIIIIIIIIIIIIIIIIIIIIIIIIIIIIIIIIIIIIM<br>MMMMMMMMMMMMMMMOOOMMMMMMMMMMMMMMMMMMMMIIIIIIIIIIIIIIIIIIIIIII<br># ApISY Number of predicted TMRs: 7
ApNEPO	MASLISGAMSSAAWTGAGMIPVFVVGLAYLRYSMYDPESRVVDVLEVRDEYDFIVVGAGSAGAVIANRLSEMQNWTVLVL<br>EAGGDETEISDVPSFVGYLQLSDMDWQYKTAPPSSDNPYCLAMVHDRCNWPRGKVLGGSSVLNAMVYVRGNQRDYDMWAA<br>AGNPGWAYADVLPYFLKSEDNRNPYLARTKYHARGGYLTVSEAPWRTPLATAFVAAGEELGYQNRDINGQYQNGFMLTQT<br>TTRRGSRCSTAKAFLRPIRLRPNIHVSMHSQVTRIHFSGGNGGSDKLRATGVTYLRNGKRRTVTARKEVILSAGAIGSPQ<br>LLMVSGVGPADHLTELGIKPVVDLKVGHNLQDHVGLGGLTFLIDDPITFKKSRFTSASVALDYIMNERGPLTSSGVEGLA<br>FVNTKYADPSGEFPDIQFHFAPSSVNSDGDQIRKITGLRDAVYNTVYKPLVNAETWTLLPLLLRPKSSGWVRLKSKNPLA<br>HPIIEPNYFAHREDVQVLVDGIRIAFNVSNTAAFRKYNSRPLLTPMPGCKKFELFSDEYWECALRHFTFTIYHPAGTCKM<br>GPDTDPDAVVDHRLRVRGIDGLRVIDASIMPNIISGNPNAPVIMIGEKGADMIKKDWSS<br>SSSSSSSSSSSSSSSSSSSSSSSSSSSSSSSSOOOOOOOOOOOOOOOOOOOOOOOOOOOOOOOOOOOOOOOOOOOOOOOO<br>OOOOOOOOOOOOOOOOOOOOOOOOOOOOOOOOOOOOOOOOOOOOOOOOOOOOOOOOOOOOOOOOOOOOOOOOOOOOOOOO<br>OOOOOOOOOOOOOOOOOOOOOOOOOOOOOOOOOOOOOOOOOOOOOOOOOOOOOOOOOOOOOOOOOOOOOOOOOOOOOOOO<br>OOOOOOOOOOOOOOOOOOOOOOOOOOOOOOOOOOOOOOOOOOOOOOOOOOOOOOOOOOOOOOOOOOOOOOOOOOOOOOOO<br>OOOOOOOOOOOOOOOOOOOOOOOOOOOOOOOOOOOOOOOOOOOOOOOOOOOOOOOOOOOOOOOOOOOOOOOOOOOOOOOO<br>OOOOOOOOOOOOOOOOOOOOOOOOOOOOOOOOOOOOOOOOOOOOOOOOOOOOOOOOOOOOOOOOOOOOOOOOOOOOOOOO<br>OOOOOOOOOOOOOOOOOOOOOOOOOOOOOOOOOOOOOOOOOOOOOOOOOOOOOOOOOOOOOOOOOOOOOOOOOOOOOOOO<br>OOOOOOOOOOOOOOOOOOOOOOOOOOOOOOOOOOOOOOOOOOOOOOOOOOOOOOOOOOOOOOOOOOOOOOOOOOOOOOOO<br>OOOOOOOOOOOOOOOOOOOOOOOOOOOOOOOOOOOOOOOOOOOOOOOOOOOOOOOOOOO<br># ApNEPO Number of predicted TMRs: 0
100164798	MSGVEVIPIGAIAGAASQVAWFLPVLVAAIAYFHYEVNDPESRIIDQPGNLILDQYDFIIVGAGSAGAVLANRLTEVEDW<br>SVLLIEAGGDETEISDVPLLAAYLQLTQLDWQYKAEPQDTACLAMKDQRCNWPRGKVLGGSSVLNYMMYVRGNQMDYDSW<br>LKQGNPGWGYNDVLYYFKKSEDNRNPYLARTPYHSMGGYLTVSEAPYKTPLADAFVRAGQEMGYDIRDINGERQTGFMIP<br>QGTIRRGARCSTAKAFLRPARLRKNLHVSINAHVTRVAINPETKVAFGVEMIKDNTRHFIRANKEVLLSAGSISSAQLLM<br>LSGIGPKNHLTEMGIPVLADLDVGKNLQDHVGLGGLAFLINKEVSLTQERVENVQTVLNYATMGDGPLTVMGGVEGLAFI<br>NTKYANQSVDTPDIELHFVSGSTNSDGGVQLWKAHGLKEEFYKAVYEPINNKDVWSAIPMLLRPKSRGEILLRSTDPSMY<br>PRILPNYLTVQEDVDTLVEGVKFVVAMSRTTPFRRYGSRLHDIPFPGCAAVPRFTDAYWECMVRHYTVTIYHPVGTAKMG<br>PEWDKTAVVDPRLRVYGIHGLRVVDASIMPTLVSANTNAPVIMIAEKAADMIKEKWLGRKR<br>SSSSSSSSSSSSSSSSSSSSSSSSSSSSSSSSOOOOOOOOOOOOOOOOOOOOOOOOOOOOOOOOOOOOOOOOOOOOOOOO<br>OOOOOOOOOOOOOOOOOOOOOOOOOOOOOOOOOOOOOOOOOOOOOOOOOOOOOOOOOOOOOOOOOOOOOOOOOOOOOOOO<br>OOOOOOOOOOOOOOOOOOOOOOOOOOOOOOOOOOOOOOOOOOOOOOOOOOOOOOOOOOOOOOOOOOOOOOOOOOOOOOOO<br>OOOOOOOOOOOOOOOOOOOOOOOOOOOOOOOOOOOOOOOOOOOOOOOOOOOOOOOOOOOOOOOOOOOOOOOOOOOOOOOO<br>OOOOOOOOOOOOOOOOOOOOOOOOOOOOOOOOOOOOOOOOOOOOOOOOOOOOOOOOOOOOOOOOOOOOOOOOOOOOOOOO<br>OOOOOOOOOOOOOOOOOOOOOOOOOOOOOOOOOOOOOOOOOOOOOOOOOOOOOOOOOOOOOOOOOOOOOOOOOOOOOOOO<br>OOOOOOOOOOOOOOOOOOOOOOOOOOOOOOOOOOOOOOOOOOOOOOOOOOOOOOOOOOOOOOOOOOOOOOOOOOOOOOOO<br>OOOOOOOOOOOOOOOOOOOOOOOOOOOOOOOOOOOOOOOOOOOOOOOOOOOOOOOOOOOOOOOOOOOOOOOOOOOOOOOO<br>OOOOOOOOOOOOOOOOOOOOOOOOOOOOOOOOOOOOOOOOOOOOOOOOOOOOOOOOOOOOO<br># ApGMC-Ox2 Number of predicted TMRs: 0
100168586	MKKIAIFGATGMTGLCTVEAALKQGLEVRALLRDPSRMPEELRKQVEVITGDVLVKEDVDKVVEGRDAIVVTLGTRNDLA<br>PTTIMSEGLRNILSSMEKNNVKIVSVCLSTFLFYDKPKVPAMFHGINDDHERMLHLLQAAESLDWIAVMPPHIAGTPSGD<br>YSVEIGSSPGRAISKYDLGKFMIECLSKPDYYKQRCGLATKVPAP<br>IIIIIIIIIIIIIIIIIIIIIIIIIIIIIIIIIIIIIIIIIIIIIIIIIIIIIIIIIIIIIIIIIIIIIIIIIIIIIII<br>IIIIIIIIIIIIIIIIIIIIIIIIIIIIIIIIIIIIIIIIIIIIIIIIIIIIIIIIIIIIIIIIIIIIIIIIIIIIIIII<br>IIIIIIIIIIIIIIIIIIIIIIIIIIIIIIIIIIIIIIIIIIIII<br># 100168586_Flavin_reductase Number of predicted TMRs: 0
100162094	MEYFLPNRCTSTVRLDGKTVVVTGCNTGIGKETATEFYKRGARVIMACRSASRTQDAIESIKNQTEGDNNVGELVFKHLE<br>LSFLASVRKCAKEILHTEKRIDILVNNAGIMMCPKTLSENGIELHLATNHLGHFLFTLLLLPRILKSAPARIINVTSLAH<br>KWGDQKMHFDDINLDKDYTPSGAYGRSKLANILFTVELAKRLNGTGVTVYAVNPGIVHTELSRYVDQTIFPGASWLYNSF<br>TKIAVKTPQQGAQTTLHCALDEKCAGESGLYYSDCKVLEPEPVAKDEEVSAQLWDTSCAFVNLEPSIDPFKPESDDVSI<br>IIIIIIIIIIIIIIIIIIIIIIIIIIIIIIIIIIIIIIIIIIIIIIIIIIIIIIIIIIIIIIIIIIIIIIIIIIIIIIII<br>IIIIIIIIIIIIIIIIIIIIIIIIIIIIIIIIIIIIIIIIIIIIIIIIIIIIIIIIIIIIIIIIIIIIIIIIIIIIIIII<br>IIIIIIIIIIIIIIIIIIIIIIIIIIIIIIIIIIIIIIIIIIIIIIIIIIIIIIIIIIIIIIIIIIIIIIIIIIIIIIII<br>IIIIIIIIIIIIIIIIIIIIIIIIIIIIIIIIIIIIIIIIIIIIIIIIIIIIIIIIIIIIIIIIIIIIIIIIIIIIIII<br># 100162094_retinol_dehydrogenase Number of predicted TMRs: 0
100162683	MLSRRCAYRSQVSRGAHGVCTMFSSDGMTCPPDADGTAACGHRRRCSGLIGCALLASEAAAHVARLCRSNPRLLSMLVQE<br>KCGDDANSSRFAHDFKTLADVLVQRVVSKRIGRQFPELEGNVYGEENDTIQNNKGQDVTIQVGETVEETLQCLTKALGDD<br>LDSAKSLAEAAHKEFESKDLNVDCYPPEEENLDLEKIGIWIDPIDSTNEYINGNVDTINEYGFHSSGLHCVTVNIGLFDK<br>YSGKPIAGVINQPFFEFDAVNGWNGRCYWAYCDGQKSLNSLPEFISCNQELVVTSNSETDAAKAALRRSGYTVATASGAG<br>YKMLCVALGIVKCYALTKDSTYAWDTCAAHAMLASQGGKACQCNTADGPLTYRPKTAGGGRAHCNAAGVIASRDPQTVDR<br>VHALLSPLHLHCSSSS<br>IIIIIIIIIIIIIIIIIIIIIIIIIIIIIIIIIIIIIIIIIIIIIIIIIIIIIIIIIIIIIIIIIIIIIIIIIIIIIIII<br>IIIIIIIIIIIIIIIIIIIIIIIIIIIIIIIIIIIIIIIIIIIIIIIIIIIIIIIIIIIIIIIIIIIIIIIIIIIIIIII<br>IIIIIIIIIIIIIIIIIIIIIIIIIIIIIIIIIIIIIIIIIIIIIIIIIIIIIIIIIIIIIIIIIIIIIIIIIIIIIIII<br>IIIIIIIIIIIIIIIIIIIIIIIIIIIIIIIIIIIIIIIIIIIIIIIIIIIIIIIIIIIIIIIIIIIIIIIIIIIIIIII<br>IIIIIIIIIIIIIIIIIIIIIIIIIIIIIIIIIIIIIIIIIIIIIIIIIIIIIIIIIIIIIIIIIIIIIIIIIIIIIIII<br>IIIIIIIIIIIIIIII<br># 100162683_inositol_polyphosphate_1-phosphatase Number of predicted TMRs: 0

Table S6: Primers used in this study.

Gene	Direction	Note	Sequence
ApRed	fwd	*Bam*HI	AGAGGGATCCGTAATGGAGAATCCTGAAGGAGA
ApRed	rev	*Xho*I	CGAGCTCGAGTCAGCTCCACACATCAGC
ApG8H	fwd	*Not*I	CCAGGCGGCCGCAATGTTTGAATTCGTCTACGAAC
ApG8H	rev	*Sac*I	CCAAGAGCTCTTAAGTATTCACTTTCCTAAATTTTAACC
ApGES	fwd	*Not*I	CGTGCGGCCGCAATGATGACTTACTCCGTTTCT
ApGES	rev	*Sac*I	ACAGAGCTCTTATTGTGACTTGGCGGAA
ApISY	fwd	*Not*I	GCAGCGGCCGCAATGATGGACGTCGTAAACG
ApISY	rev	*Sac*I	TCGGAGCTCTTACAGAATATACGGTATGATAGCT

Table S7: The iridoid pathway genes are randomly distributed throughout the *A. pisum* genome.

Gene	Scaffold (JIC 1.1.0)	Position
ApISY	2	63,420,766
ApHGO	2	11,700,000
ApG8H	2	98,800,000
ApIDS	3	81,800,000
ApGES	1	127,500,000
ApNEPO	4	36,000,000
